# Genes in loci genetically associated with polycystic ovary syndrome are dynamically expressed in human fetal gonadal, metabolic and brain tissues

**DOI:** 10.3389/fendo.2023.1149473

**Published:** 2023-05-08

**Authors:** Rafiatu Azumah, Katja Hummitzsch, Richard A. Anderson, Raymond J. Rodgers

**Affiliations:** ^1^ Robinson Research Institute, School of Biomedicine, The University of Adelaide, Adelaide, SA, Australia; ^2^ Medical Research Council Centre for Reproductive Health, Queen’s Medical Research Institute, University of Edinburgh, Edinburgh, United Kingdom

**Keywords:** fetus, ovary, testis, kidney, liver, heart, brain, PCOS (polycystic ovarian syndrome)

## Abstract

**Background:**

Polycystic ovary syndrome (PCOS) is a heterogeneous disorder, affecting around 10% of women of reproductive age, with infertility, depression or anxiety, obesity, insulin resistance and type 2 diabetes as risk factors. The cause of PCOS is not known but there is a predisposition to developing PCOS in adult life that arises during fetal or perinatal life. PCOS also has a genetic predisposition and a number of genetic loci associated with PCOS have been identified. These loci contain 25 candidate genes which are currently being studied to define the syndrome. Although the name PCOS suggests a syndrome of the ovary, PCOS has also been associated with the central nervous system and other organ systems in the body due to the wide variety of symptoms it presents.

**Methods:**

Here, we examined the expression patterns of PCOS candidate genes in gonadal (ovary and testis), metabolic (heart, liver and kidney) and brain (brain and cerebellum) tissues during the first half of human fetal development and postnatally until adulthood using public RNA sequencing data. This study is an initial step for more comprehensive and translational studies to define PCOS.

**Results:**

We found that the genes were dynamically expressed in the fetal tissues studied. Some genes were significantly expressed in gonadal tissues, whilst others were expressed in metabolic or brain tissues at different time points prenatally and/or postnatally. *HMGA2*, *FBN3* and *TOX3* were highly expressed during the early stages of fetal development in all tissues but least during adulthood. Interestingly, correlation between expression of *HMGA2/YAP1* and *RAD50/YAP1* were significant in at least 5 of the 7 fetal tissues studied. Notably, *DENND1A, THADA, MAPRE1, RAB5B, ARL14EP, KRR1, NEIL2* and *RAD50* were dynamically expressed in all postnatal tissues studied.

**Conclusions:**

These findings suggest that these genes have tissue- or development-specific roles in multiple organs, possibly resulting in the various symptoms associated with PCOS. Thus the fetal origin of a predisposition to PCOS in adulthood could arise *via* the effects of PCOS candidate genes in the development of multiple organs.

## Introduction

Polycystic ovary syndrome (PCOS) is a heterogeneous disorder that affects 10% women of reproductive age of which 72% suffer infertility due to anovulation (1). The disorder has become a public health concern presenting long-term complications in women, notwithstanding challenges of diagnosis to clinicians and researchers. The syndrome presents varying symptoms ranging from endocrine features including hyperandrogenism (hirsutism, acne, alopecia); reproductive features including menstrual irregularities and infertility; metabolic features such as insulin resistance, obesity, hyperinsulinemia, type 2 diabetes mellitus; and cardiovascular features including atherogenic dyslipidaemia, a prothrombotic state, elevated blood pressure and increased circulation proinflammatory markers (2–9). The syndrome also affects the psychological health of patients causing anxiety and sleep disturbances as well as sleep disorders (10). Aside from the challenges of delayed diagnosis and lack of treatment options, these symptoms collectively worsen the psychological health of PCOS women.

Some of the metabolic symptoms of PCOS have also been observed in male offspring of PCOS mothers in both human and animal studies (11–16). However, the cause of the syndrome still remains unclear. The genetic and fetal origins of the disorder have become the focus of current studies. Previous studies have shown that PCOS candidate genes including those in loci identified in GWAS and microsatellite genotyping are dynamically expressed in human and bovine fetal ovaries (17–19). Also, these genes are co-expressed with genes involved in mitochondrial function, stromal expansion and steroidogenesis during fetal ovary development (18). Even though, it is not clear how these genes further regulate canonical pathways during fetal development leading to PCOS predisposition later in life, numerous theories in the literature have associated these pathways to the aetiology of PCOS. Interestingly, these candidate genes were not differentially expressed in the ovaries of adult women with PCOS when compared with controls (19), further supporting the possible fetal origin of the syndrome.

Although the name PCOS suggests a syndrome of the ovary, the disorder presents symptoms associated with other organs of the body. The majority of PCOS studies have focussed on the role of the ovary in understanding the disorder, however, most of these studies have increased the conundrum surrounding the syndrome. Currently, animal studies, including genetic knock-out studies, are focussing on the role of genes in or near loci associated with PCOS; towards delineating the disorder. However, studies delineating the expression patterns of these candidate genes during fetal and adult development in different tissues/organs, other than the ovary, are lacking. Thus, this study seeks to delineate the expression patterns of genes in loci associated with PCOS (17–19) in gonadal, metabolic and brain tissues during fetal development as well as postnatally until adulthood using publicly available human RNA sequencing data. For the purpose of presentation and discussion, the PCOS candidate genes were grouped based on their known basic functions; DNA/RNA regulation/processing (*HMGA2*, *TOX3*, *GATA4*, *YAP1*, *ZBTB16*, *IRF1*, *NEIL2*, *RAD50*, *KRR1*), cellular functions (*RAB5B, ARL14EP, DENND1A, THADA, MAPRE1*), enzymatic reactions (*C9orf3/AOPEP, SUOX, SUMO1P1*), cell surface receptors (*ERBB3, ERBB4, PLGRKT*), extracellular matrix regulation (*FBN3*), metabolism (*INSR, FDFT1*), and reproduction (*FSHB*, *FSHR*, *LHCGR*, *AR*, *AMH*).

## Materials and methods

We analysed normalised human RNA-sequencing data (counts per million) deposited in Array Express (E-MTAB-6814) from the ‘*Gene expression across mammalian organ development*’ project, which sampled seven organs collected from males and females. The prenatal samples in that project were provided by the MRC-Wellcome Trust Human Developmental Biology Resource based in the United Kingdom. They were from elective abortions with normal karyotypes. Postnatal samples were provided by the NICHD Brain and Tissue Bank for Developmental Disorders at the University of Maryland, USA, and by the Chinese Brain Bank Center in Wuhan, China. They originated from individuals with diverse causes of death that, given the information available, were not associated with the organ sampled. The patient information provided in this project was gender and age or developmental status (**Tables S1–S3**). The status regarding any PCOS is unknown but it is possible that some samples are from such women. The organs in this project represent the three germ layers: the ectoderm consisting of brain (forebrain/cerebrum) and cerebellum (hindbrain/cerebellum); the mesoderm of heart, kidney, ovary and testis; and the endoderm of the liver (20). The expression of PCOS candidate genes was studied in all 7 tissues in fetuses 4-20 weeks post conception (wpc) and from birth till adulthood. Fetal ovary samples were available only up to 18 wpc and no postpartum samples were available, while kidney samples were collected only up to 8 years. Postnatally, we grouped the samples as prepubertal (from birth till 9 years), pubertal (13-19 years) and adulthood (each decade until 65 years of age). In testis samples, 13-14 years were considered as early puberty, and 15-19 as late puberty as grouped in the original study (20).

The tissues were grouped according to function; gonads (ovary and testis), metabolic tissues (liver, kidney, and heart), and brain tissues (brain and cerebellum). Expression data of PCOS candidate genes studied previously (17–19) were extracted from normalised data and further analysed. The possible influence of transcriptional and post-transcriptional mechanisms such as mRNA stability/degradation, storage in stress granules, translational control was not the focus of this study. Specifically, time-course scatterplots were generated for each gene as grouped for all samples available using ggplot2 package in R (21). **Tables S1–3** show the sex and specific time points (gestational age/years postnatally) of samples plotted for each gene. Pearson’s correlation was then carried out for each fetal tissue separately using IBM SPSS Statistics for Windows, version 25 (IBM Corp., Armonk, NY, USA). The correlation of each gene with others within each tissue was then compared with that in the other tissues and the output collated based on all significant correlations (*P* < 0.01). No further statistical analysis was conducted as this study, from a basic science perspective, provides an initial set of results for more comprehensive and translational studies.

## Results

### Expression in fetal tissues

Gene expression in the context of this study refers to the steady-state mRNA levels measured. The expression of PCOS candidate genes was studied in 7 fetal tissues (ovary, testis, heart, liver, kidney, brain and cerebellum) from early till mid gestation (4-20 wpc). All genes were dynamically expressed across all tissues, with few exceptions. Although there were fewer female samples than males, there was no difference in gene expression observed between the two sexes in the same tissues, excluding the gonads. We compared gene expression among different tissues and found that some genes were significantly expressed in gonadal tissues, whilst others were expressed in the metabolic or brain tissues at different time points prenatally and/or postnatally.

**Figure 1:**
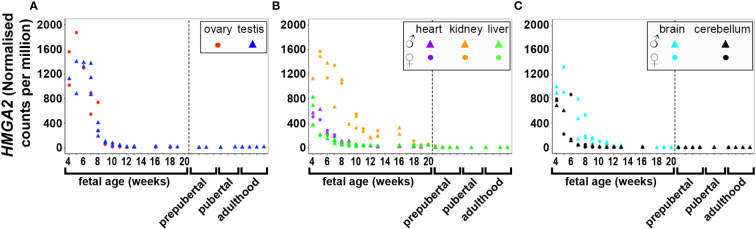
Expression of *HMGA2* in gonadal **(A)**, metabolic **(B)** and brain tissues **(C)** during the first half of fetal development and postnatally. Short dashes distinguish fetal samples from postnatal ones.

**Figure 2:**
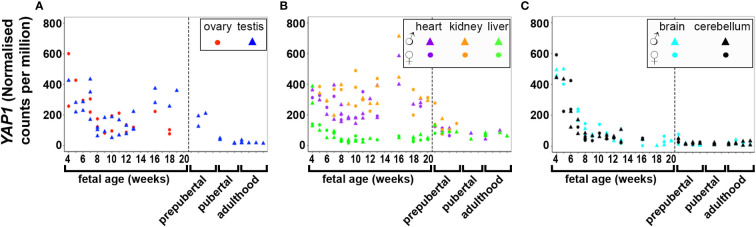
Expression of *YAP1* in gonadal **(A)**, metabolic **(B)** and brain tissues **(C)** during the first half of fetal development and during lifetime. Short dashes distinguish fetal samples from postnatal ones.

**Figure 3:**
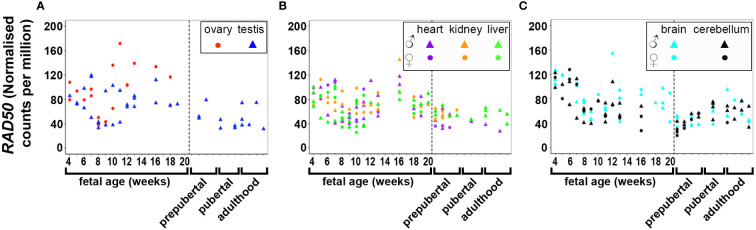
Expression of *RAD50* in gonadal **(A)**, metabolic **(B)** and brain tissues **(C)** during the first half of fetal development and during lifetime. Short dashes distinguish fetal samples from postnatal ones.

DNA and RNA regulation/processing genes (*HMGA2*, *TOX3*, *GATA4*, *YAP1*, *ZBTB16*, *IRF1*, *NEIL2*, *RAD50*, *KRR1*) were dynamically expressed across gestation. *HMGA2* was highly expressed at 4 weeks and expression decreased significantly till mid gestation in all tissues (**Figure 1**). This expression pattern was also observed in *YAP1* (**Figure 2**) and *ZBTB16* (**Figure S1A**) in both brain tissues. *IRF1* and *GATA4* were not detected in the brain and cerebellum (**Figures S1B, C**). Levels of all other genes in this group are very consistently expressed in the two brain tissues during the 20 weeks. The expression of *YAP1* also declined in the liver and ovary, whereas it had a U-shaped distribution in the testis and was highly expressed throughout gestation in heart and kidney (**Figure 2**). Furthermore, *GATA4* expression increased significantly until 13 weeks in gonadal tissues and levels drastically decreased thereafter (**Figure S1C**). A similar steep increase in expression with a decline after 10 weeks was observed for *TOX3* in the fetal ovary, whereas levels in the testis were always low (**Figure S1D**). In metabolic tissues, *GATA4* was highly expressed in heart tissues throughout gestation, whereas the expression in the liver was highest at 4 weeks and then declined, however, only low levels of expression were observed in fetal kidneys. *TOX3*, on the other hand, was least expressed in metabolic tissues with a slight increase in expression in kidney tissues towards mid-gestation (**Figure S1D**). *ZBTB16* expression was low and remained relatively constant in most of the fetal tissues (**Figure S1A**). *IRF1* levels increased slightly as the fetus developed in both gonadal and metabolic tissues (**Figure S1B**). *NEIL2* was moderately expressed in the metabolic and brain tissues, however, the levels increased slightly after 14 weeks in the brain tissues (**Figure S1E**). In addition, *NEIL2* expression increased significantly in the gonadal tissues, especially the ovary, as the fetus developed. *RAD50* was expressed highest at 12 weeks in the ovary, but remained relatively constant in all tissues studied (**Figure 3**). *KRR1* was dynamically expressed in all tissues with levels remaining relatively constant throughout the first half of gestation (**Figure S1F**).

Cell function genes (*RAB5B, ARL14EP, DENND1A, THADA, MAPRE1*), were expressed at relatively constant levels in most tissues with very few exceptions (**Figure S2**). In brain tissues, expression levels of *THADA* and *MAPRE1* decreased significantly towards 20 weeks. The expression of the genes for the enzymes *C9orf3/AOPEP, SUOX* and *SUMO1P1* was relatively low or absent in most fetal tissues (**Figure S3**).

Cell surface receptor genes (*ERBB3, ERBB4, PLGRKT*) were dynamically expressed in the tissues studied. *ERBB3* was least expressed in the ovary, heart and brain (brain and cerebellum) tissues, but highly in testis from week 7 up until week 14 before declining. *ERBB3* was also highly expressed in the fetal kidney and liver throughout the 20 weeks (**Figure S4A**). Although, *ERBB4* was least expressed in the liver and both gonadal tissues, it was highly expressed in the kidney, heart, and both brain tissues during early to mid-stage fetal development (**Figure S4B**). *PLGRKT* was evenly expressed in all fetal tissues throughout the 20 weeks of gestation (**Figure S4C**). Like *GATA4*, *FBN3* levels increased until 8 weeks and levels decreased thereafter in gonadal, kidney and heart tissues (**Figure S5**). However, *FBN3* was least expressed in the liver and heart tissues whilst levels remained relatively constant in the brain tissues.

Metabolic genes (*INSR, FDFT1*) were expressed significantly in all tissues during fetal development. *INSR* expression remained relatively constant in all fetal tissues throughout the 20 weeks of gestation (**Figure S6A**). *FDFT1* was expressed higher in the first weeks of fetal liver development, but decreased significantly thereafter. In the fetal testis, *FDFT1* showed a steep increase in expression between 8-13 weeks before dramatically declining towards 20 weeks. *FDFT1* was least expressed in the kidney and heart during the early stages of fetal development (**Figure S6B**). Notably, genes involved in reproduction (*FSHB*, *FSHR*, *LHCGR*, *AR*, *AMH*) were not expressed in most of the fetal tissues before mid-gestation, as expected. *FSHB* was only detected in three kidney and one cerebellum samples during mid-gestation, but not in all other fetal samples throughout the 20 weeks (**Figure S7B**). *FSHR, LHCGR* and *AMH* levels showed a steep increase after 7-8 weeks gestation in the fetal testis, whereas levels remained relatively low in the fetal ovary (**Figures S7B, C, E**). An increasing expression of *FSHR* and *AR* were observed in ovary tissues at low levels till mid-gestation (**Figure S7**).

### Relationships of gene expression in fetal tissues

Pearson’s correlation of candidate genes with each other as well as with gestational age was carried out for each tissue separately and the outcome of all significant correlations (*P* < 0.01) for all tissues collated into a table for each gene. All PCOS candidate genes showed a significant (*P* < 0.01) correlation with gestational age (days) in at least one tissue studied except for *FSHB, SUOX* and *SUMO1P1* (**Table 1**). Selected gene expression relationships are reported here, however, detailed data on other genes not mentioned here can be found in supplementary tables (**Tables S4–28**). Interestingly, the correlation of *HMGA2/YAP1* and *RAD50/YAP1* were significant in at least 5 of the 7 fetal tissues studied.

**Table 1:** Pearson’s correlation coefficients (R) between PCOS candidate genes mRNA expression levels and gestational age across individual tissues.

Genes	Ovary	Testis	Heart	Kidney	Liver	Cerebellum	Brain	
*FSHB*	**-**	***-***	**-**	0.46^a^	–	0.35^a^	–	
*FSHR*	0.24	0.16	-0.41^a^	-0.50^b^	-0.28	-0.30	-0.45^b^	2-
*LHCGR*	-0.42	0.20	-0.03	-0.12	-0.46^b^	-0.37^a^	-0.61^c^	2-
*AR*	0.51^a^	0.85^d^	0.16	0.09	0.64^d^	-0.58^c^	-0.75^d^	2+, 2-
*AMH*	-0.17	0.00	-0.33	-0.56^b^	-0.25	0.04	-0.34	1-
*INSR*	-0.14	0.36	0.29	0.31	0.52^b^	0.20	0.74^d^	2+
*FDFT1*	0.20	0.17	-0.26	-0.57^b^	-0.42^a^	-0.72^d^	-0.14	2-
*ERBB3*	-0.31	0.05	-0.39^a^	0.52^b^	-0.04	-0.34	-0.38^a^	1+
*ERBB4*	-0.61^b^	-0.47^a^	0.12	0.79^d^	-0.29	0.23	0.29	1+, 1-
*PLGRKT*	-0.72^c^	-0.32	0.53^b^	0.52^b^	0.14	-0.48^b^	-0.15	2+, 2-
*HMGA2*	-0.77^c^	-0.75^d^	-0.69^d^	-0.85^d^	-0.55^c^	-0.64^d^	-0.72^d^	7-
*TOX3*	0.34	-0.50^b^	-0.31	0.68^d^	-0.51^b^	-0.60^c^	-0.07	1+, 3-
*GATA4*	0.45	-0.15	-0.51^b^	-0.53^b^	-0.66^d^	-0.03	-0.22	3-
*YAP1*	-0.65^b^	-0.02	0.21	0.12	-0.47^b^	-0.72^d^	-0.74^d^	4-
*ZBTB16*	0.43	0.62^c^	0.43^a^	0.25	0.15	-0.66^d^	-0.57^c^	1+, 2-
*IRF1*	0.69^b^	0.84^d^	0.11	0.67^c^	0.82^d^	0.54^b^	0.04	5+
*NEIL2*	0.75^c^	0.31	-0.42^a^	-0.56^b^	0.27	0.75^d^	0.67^d^	3+, 1-
*RAD50*	0.31	0.04	0.01	-0.04	0.05	-0.61^c^	-0.28	1-
*KRR1*	0.01	0.29	0.34	0.30	0.36^a^	-0.58^c^	-0.28	1-
*RAB5B*	-0.04	0.27	0.33	0.54^b^	0.32	0.06	0.25	1+
*ARL14EP*	-0.39	0.30	0.53^b^	0.32	-0.03	-0.02	0.30	1+
*DENND1A*	0.33	-0.14	-0.35^a^	0.01	-0.22	0.66^d^	0.09	1+
*THADA*	0.21	-0.12	-0.11	0.01	-0.36^a^	-0.79^d^	-0.78^d^	2-
*MAPRE1*	0.06	-0.19	-0.41^a^	-0.61^c^	0.16	-0.78^d^	-0.69^d^	3-
*AOPEP*	0.02	0.11	0.28	0.57^b^	-0.20	-0.41^a^	-0.65^d^	1+, 1-
*SUOX*	-0.26	-0.41^a^	-0.27	0.12	-0.34^a^	0.16	-0.13	
*SUMO1P1*	0.09	0.10	-0.18	0.06	0.00	–	0.22	
*FBN3*	-0.44	-0.73^d^	-0.77^d^	-0.73^d^	-0.46^b^	-0.17	0.43^a^	4-

Positive and negative correlations are marked in pink and blue, respectively. The colour intensity corresponds with the strength of the correlation. P-values: ^a^ < 0.05; ^b^ < 0.01; ^c^ < 0.001; ^d^ < 0.0001. Tissues with P < 0.01 were regarded as significant. ^Ϯ^Number of organs with significant (P < 0.01), positive (+) or negative (-) correlations. (-) for no gene expression.

While *HMGA2* significantly correlated negatively with gestational age in all tissues studied, *IRF1* correlated with gestational age in 5 tissues including gonads and cerebellum but not heart and brain tissues (**Table 1**). *FBN3* expression correlated negatively with gestational age in testis and all metabolic tissues while *GATA4* correlated negatively with gestational age in all metabolic tissues. *AR* correlated positively with gestational age in the gonads and the liver but negatively in brain tissues. *LHCGR, YAP1, ZBTB16, THADA*, and *MAPRE1* correlated negatively with gestational age in brain tissues but positively with *NEIL2* (**Table 1**).

Interestingly, RNA/DNA regulation/processing genes correlated with each other and other candidate gene significantly in at least 3 tissues studied. For instance, *HMGA2* expression significantly correlated positively with *FSHR, ERBB3/4, TOX3, YAP1, THADA, MAPRE1*, and *FBN3*, but negatively with *INSR*, and *IRF1* in at least 3 tissues studied (**Table 2**)*. YAP1* expression significantly correlated positively with *FSHR, LHCGR, ERBB4, HMGA2, RAD50, KRR1, ARL14EP, THADA*, and *MAPRE1*, but negatively with *INSR* and *DENND1A* in at least 3 tissues studied (**Table 3**)*. ZBTB16* expression significantly correlated positively with *AR, RAD50, THADA* and *MAPRE1* in at least 3 tissues studied (**Table S6**)*. RAD50* expression significantly correlated with *THADA* in all tissues, with *YAP1, KRR1, MAPRE1* in 6 tissues and with *AR, AMH, ZBTB16, RAB5B, ARL14EP, DENND1A* and *AOPEP* in at least 3 tissues (**Table 4**). *KRR1* expression significantly correlated positively with *YAP1, ZBTB16, RAD50, THADA* and *MAPRE1* but negatively with *AMH* and *SUOX* in at least 3 tissues (**Table S9**).

**Table 2:** Pearson’s correlation coefficients (R) between PCOS candidate genes mRNA expression levels and *HMGA2* across individual tissues.

Genes	Ovary	Testis	Heart	Kidney	Liver	Cerebellum	Brain	
*FSHB*	* *-	-	-	-0.34	-	-0.09	-	
*FSHR*	-0.61^b^	-0.37	0.67^d^	0.64^c^	0.82^d^	0.59^c^	0.45^b^	5+, 1-
*LHCGR*	0.52^a^	-0.58^b^	0.18	0.13	0.41^a^	0.68^d^	0.68^d^	2+, 1-
*AR*	-0.31	-0.49^a^	-0.03	0.19	-0.050	0.92^d^	0.85^d^	2+
*AMH*	-0.16	-0.55^b^	-0.12	0.53^b^	-0.03	-0.330	-0.044	1+, 1-
*INSR*	-0.16	-0.71^d^	-0.33	-0.22	-0.61^d^	-0.42^a^	-0.65^d^	3-
*FDFT1*	-0.07	-0.50^b^	0.75^d^	0.57^b^	0.15	0.41^a^	-0.176	2+, 1-
*ERBB3*	0.05	-0.37	0.82^d^	-0.68^d^	0.10	0.68^d^	0.60^c^	3+, 1-
*ERBB4*	0.77^c^	0.60^c^	0.11	-0.74^d^	0.78^d^	-0.32	-0.22	3+, 1-
*PLGRKT*	0.63^b^	-0.09	-0.60^c^	-0.42^a^	-0.22	0.19	-0.10	1+, 1-
*HMGA2*	*	*	*	*	*	*	*	
*TOX3*	-0.59^a^	0.86^d^	0.58^c^	-0.54^b^	0.62^d^	0.11	0.05	3+, 1-
*GATA4*	-0.82^d^	-0.25	0.33	0.70^d^	0.94^d^	-0.14	-0.08	2+
*YAP1*	0.86^d^	0.53^b^	0.20	0.06	0.97^d^	0.96^d^	0.92^d^	5+
*ZBTB16*	-0.40	-0.26	-0.14	-0.27	0.20	0.86^d^	0.76^d^	2+
*IRF1*	-0.62^b^	-0.72^d^	0.19	-0.71^d^	-0.42^b^	0.02	0.25	4-
*NEIL2*	-0.78^c^	-0.60^b^	0.53^b^	0.67^c^	-0.17	-0.30	-0.34	2+, 2-
*RAD50*	-0.20	0.29	0.35^a^	0.28	0.39^a^	0.67^d^	0.53^b^	2+
*KRR1*	-0.12	0.09	-0.05	-0.09	-0.08	0.54^b^	0.50^b^	2+
*RAB5B*	0.33	-0.05	0.12	-0.44^a^	0.24	0.11	-0.03	
*ARL14EP*	0.48^a^	-0.01	-0.34	-0.11	0.35^a^	-0.11	-0.18	
*DENND1A*	-0.70^b^	0.09	0.62^c^	-0.05	0.18	-0.38^a^	0.01	1+, 1-
*THADA*	-0.23	0.21	0.53^b^	0.02	0.52^b^	0.87^d^	0.89^d^	4+
*MAPRE1*	-0.10	-0.03	0.54^c^	0.61^c^	0.26	0.50^b^	0.58^c^	4+
*AOPEP*	-0.06	-0.20	-0.59^c^	-0.58^b^	-0.25	0.67^d^	0.85^d^	2+, 2-
*SUOX*	0.12	0.20	-0.04	-0.23	-0.06	-0.01	0.26	
*SUMO1P1*	-0.45	-0.08	0.35^a^	-0.01	-0.13	–	-0.10	
*FBN3*	-0.02	0.75^d^	0.87^d^	0.73d	0.95^d^	0.26	-0.01	4+

Positive and negative correlations are marked in pink and blue, respectively. The colour intensity corresponds with the strength of the correlation. P-values: a < 0.05; b < 0.01; c < 0.001; d < 0.0001. Tissues with P < 0.01 were regarded as significant. ^Ϯ^Number of organs with significant (P < 0.01), positive (+) or negative (-) correlations. ‘-’ and ‘*’ were for no gene expression and correlation between same gene, respectively.

**Table 3:** Pearson’s correlation coefficients (R) between PCOS candidate genes mRNA expression levels and *YAP1* across individual tissues.

Genes	Ovary	Testis	Heart	Kidney	Liver	Cerebellum	Brain	
*FSHB*	-	-	-	-0.19	-	-0.12	-	
*FSHR*	-0.45	-0.35	0.41^a^	0.01	0.85^d^	0.62^c^	0.63^b^	3+
*LHCGR*	0.71^c^	-0.57^b^	0.21	0.05	0.41^a^	0.69^d^	0.80^d^	3+, 1-
*AR*	-0.15	0.27	0.40^a^	0.11	0.01	0.94^d^	0.93^d^	2+
*AMH*	-0.39	-0.86^d^	-0.60^c^	-0.46^a^	-0.11	-0.40^a^	-0.10	2-
*INSR*	-0.10	-0.58^b^	0.10	-0.07	-0.50^b^	-0.39^a^	-0.68^b^	3-
*FDFT1*	0.17	-0.53^b^	0.36^a^	-0.05	0.15	0.52^b^	-0.09	1+, 1-
*ERBB3*	0.22	-0.44^a^	0.37^a^	0.03	0.09	0.65^d^	0.73^c^	2+
*ERBB4*	0.93^d^	0.67^c^	0.82^d^	0.49^b^	0.83^d^	-0.25	-0.08	5+
*PLGRKT*	0.51^a^	-0.59^b^	-0.03	0.54^b^	-0.24	0.25	-0.21	1+, 1-
*HMGA2*	0.86^d^	0.53^b^	0.20	0.06	0.97^d^	0.96^d^	0.92^d^	5+
*TOX3*	-0.31	0.63^c^	0.04	0.31	0.67^d^	0.25	0.16	2+
*GATA4*	-0.77^c^	-0.60^b^	-0.08	-0.04	0.86^d^	-0.10	-0.05	1+, 2-
*YAP1*	*	*	*	*	*	*	*	
*ZBTB16*	-0.10	0.40^a^	0.22	-0.42^a^	0.30	0.87^d^	0.92^d^	2+
*IRF1*	-0.56^a^	-0.16	0.14	-0.42^a^	-0.36^a^	-0.05	0.17	
*NEIL2*	-0.61^b^	-0.63^c^	0.13	-0.42^a^	-0.14	-0.35^a^	-0.35	2-
*RAD50*	0.18	0.72^d^	0.83^d^	0.90^d^	0.52^b^	0.75^d^	0.58^c^	6+
*KRR1*	0.23	0.61^c^	0.86^d^	0.91^d^	0.07	0.65^d^	0.57^c^	5+
*RAB5B*	0.50^a^	0.40^a^	0.64^d^	-0.12	0.36^a^	0.18	0.06	1+
*ARL14EP*	0.72^c^	0.50^a^	0.56^c^	0.84^d^	0.47^b^	0.00	-0.20	4+
*DENND1A*	-0.60^b^	-0.04	0.18	-0.57^b^	0.18	-0.53^b^	0.00	3-
*THADA*	0.13	0.36	0.74^d^	0.67^c^	0.53^c^	0.93^d^	0.94^d^	5+
*MAPRE1*	0.22	-0.06	0.60^c^	0.47^a^	0.37^a^	0.63^d^	0.62^c^	3+
*AOPEP*	-0.26	-0.34	-0.47^b^	-0.14	-0.32	0.63^d^	0.91^d^	2+, 1-
*SUOX*	-0.04	-0.37	-0.60^c^	-0.51^b^	-0.15	-0.08	0.27	2-
*SUMO1P1*	-0.42	0.05	0.40^a^	-0.06	-0.14	–	-0.16	
*FBN3*	-0.20	0.13	-0.02	-0.29	0.92^d^	0.08	0.02	1+

Positive and negative correlations are marked in pink and blue, respectively. The colour intensity corresponds with the strength of the correlation. P-values: a < 0.05; b < 0.01; c < 0.001; d < 0.0001. Tissues with P < 0.01 were regarded as significant. ^Ϯ^Number of organs with significant (P < 0.01), positive (+) or negative (-) correlations. ‘-’ and ‘*’ were for no gene expression and correlation between same gene, respectively.

**Table 4:** Pearson’s correlation coefficients (R) between PCOS candidate genes mRNA expression levels and *RAD50* across individual tissues.

Genes	Ovary	Testis	Heart	Kidney	Liver	Cerebellum	Brain	
*FSHB*	*-*	–	–	-0.19	–	-0.16	–	
*FSHR*	0.10	0.21	0.57^c^	0.31	0.32	0.41^a^	0.38^a^	1+
*LHCGR*	0.05	0.00	0.29	0.20	0.04	0.39^a^	0.37^a^	
*AR*	0.59^b^	0.21	0.42^a^	0.35	0.27	0.69^d^	0.51^b^	3+
*AMH*	-0.67^b^	-0.56^b^	-0.64^d^	-0.33	-0.55^c^	-0.6^c^	-0.44^a^	5-
*INSR*	-0.06	-0.10	0.01	0.00	-0.14	0.17	0.09	
*FDFT1*	0.42	0.03	0.47^b^	0.05	0.21	0.36^a^	-0.13	1+
*ERBB3*	0.32	-0.01	0.50^b^	-0.21	0.12	0.40^a^	0.42^a^	1+
*ERBB4*	0.11	0.53^b^	0.66^d^	0.30	0.31	0.25	0.02	2+
*PLGRKT*	-0.39	-0.50^b^	-0.17	0.31	-0.08	-0.02	-0.43^a^	1-
*HMGA2*	-0.20	0.29	0.35^a^	0.28	0.40^a^	0.67^d^	0.53^b^	2+
*TOX3*	0.55^a^	0.53^b^	0.21	0.16	0.37^a^	0.50^b^	0.21	2+
*GATA4*	0.03	-0.09	0.01	0.24	0.30	0.11	-0.18	
*YAP1*	0.18	0.72^d^	0.83^d^	0.90^d^	0.52^b^	0.75^d^	0.58^c^	6+
*ZBTB16*	0.71^c^	0.09	0.31	-0.42^a^	0.70^d^	0.69^d^	0.56^c^	4+
*IRF1*	0.41	0.14	0.17	-0.51^b^	-0.02	-0.14	-0.04	1-
*NEIL2*	0.48^a^	-0.23	0.18	-0.18	0.01	-0.50^b^	-0.29	
*RAD50*	*	*	*	*	*	*	*	
*KRR1*	0.52^a^	0.63^c^	0.71^d^	0.85^d^	0.44^b^	0.90^d^	0.70^d^	6+
*RAB5B*	0.59^a^	0.77^d^	0.80^d^	-0.22	0.86^d^	0.54^b^	0.49^b^	5+
*ARL14EP*	0.36	0.53^b^	0.30	0.83^d^	0.37^a^	0.47^b^	0.41^a^	3+
*DENND1A*	0.03	0.11	0.44^b^	-0.62^c^	0.44^b^	-0.45^b^	0.03	2+, 2-
*THADA*	0.83^d^	0.60^b^	0.90^d^	0.56^b^	0.64^d^	0.82^d^	0.65^d^	7+
*MAPRE1*	0.78^c^	0.51^b^	0.69^d^	0.48^a^	0.75^d^	0.65^d^	0.52^b^	6+
*AOPEP*	-0.58^a^	-0.59^b^	-0.63^d^	-0.41^a^	-0.80^d^	0.38^a^	0.45^b^	2+, 2-
*SUOX*	-0.12	-0.40^a^	-0.58^c^	-0.54^b^	-0.29	-0.37^a^	-0.13	2-
*SUMO1P1*	-0.18	0.15	0.33	0.05	-0.08	–	-0.24	
*FBN3*	-0.62^b^	-0.10	0.14	-0.17	0.33^a^	-0.16	0.20	1-

Positive and negative correlations are marked in pink and blue, respectively. The colour intensity corresponds with the strength of the correlation. P-values: a < 0.05; b < 0.01; c < 0.001; d < 0.0001. Tissues with P < 0.01 were regarded as significant. ^Ϯ^Number of organs with significant (P < 0.01), positive (+) or negative (-) correlations. ‘-’ and ‘*’ were for no gene expression and correlation between same gene, respectively.

More so, genes involved in cell function (*RAB5B, ARL14EP, DENND1A, THADA*, and *MAPRE1)* significantly correlated with *RAD50* in at least 4 tissues (**Tables S10–14**). *THADA* and *MAPRE1* significantly correlated positively with each other in 5 tissues and both genes correlated positively with *FDFT1, HMGA2, ZBTB16, KRR1*, and *THADA* in at least 3 tissues. More so, *ERBB3* significantly correlated with *FSHR, LHCGR, HMGA2, NEIL2, THADA*, and *AOPEP* in at least 3 tissues (**Table S18**). *ERBB4* significantly correlated with *HMGA2, TOX3, YAP1*, and *RAB5B* in at least 3 tissues studied (**Table S19**).

In testis tissues, *INSR* and *FDFT1* expression significantly correlated with each other positively; both genes also significantly correlated positively with *FSHR, LHCGR, AMH, GATA4*, and *NEIL2*, but negatively with *HMGA2* and *YAP1* (**Tables S22**, **S23**). *FSHB* expression did not correlate with other reproductive genes, but correlated significantly with *ZBTB16* and *IRF1* in the kidney (**Table S24**). *FSHR* significantly correlated positively with other genes including *LHCGR, AR, ERBB3, HMGA2*, and *GATA4* in at least 4 tissues (**Table S25**). *LHCGR* expression significantly correlated positively with *AMH, INSR, FDFT1, ERBB3, NEIL2*, and *MAPRE1*, but negatively with *HMGA2* and *YAP1* in the testis (**Table S26**). Detailed correlation relationships on other genes are in **Supplementary Tables S4–S28**.

### Postnatal gene expression

The expression of PCOS candidate genes was studied in 5 tissues (testis, heart, liver, brain, and cerebellum) from birth till adulthood and in kidney samples till 8 years old. No data from postnatal ovary samples were available. We compared the levels of gene expression in the fetal tissues to those in the corresponding postnatal tissues. We found that *RAD50* (**Figure 3**), *KRR1, NEIL2* (**Figure S4**), and *DENND1A, THADA, MAPRE1, RAB5B*, and *ARL14EP* (**Figure S5**) were dynamically expressed in all postnatal tissues studied.

*HMGA2* (**Figure 1**) and *TOX3* (**Figure S1D**, except for prepubertal kidney), were not expressed in any tissue postnatally. *RAD50, NEIL2*, and *KRR1* (**Figure 3** and **Figures S1E, F** respectively) were expressed in all tissues studied. *YAP1* expression was high in testis, kidney and heart during fetal life, but decreased to very low levels towards adulthood (**Figure 2**). The expression levels in liver, brain and cerebellum remained low postnatally similar to fetal levels. *ZBTB16* expression levels in the metabolic tissues increased during the prepubertal stage and then slowly declined towards adulthood (**Figure S1A**), whereas levels in the two brain tissues increased with age. Expression levels in the testis remained relatively constant postnatally at levels similar to those of mid gestation. *IRF1* expression increased dramatically from birth until adulthood in all three metabolic tissues (**Figure S1B**). *GATA4* was only significantly expressed in the heart tissues and slightly in testis samples after puberty, whereas all other tissues showed low to nil expression (**Figure S1C**). Even the expression levels in the brain tissues were slightly higher after birth and throughout lifetime. On the other hand, in testis *IRF1* expression was dramatically higher during prepuberty, and then declined again towards adulthood to levels comparable to mid-gestation.

All genes involved in cell function (*RAB5B, ARL14EP, DENND1A, THADA, MAPRE1*) were dynamically expressed in all tissues studied (**Figure S2**). Notably, *RAB5B* levels increased slightly from birth till the end of puberty in the brain tissues where they remained relatively constant thereafter (**Figure S2A**). *ARL14EP* and *DENND1A* levels remained relatively constant in all tissues postnatally and at similar levels as during fetal life (**Figures S2B, C**). *THADA* and *MAPRE1* levels decreased slightly after birth in all tissues compared to fetal life (**Figures S2D, E**).

*C9orf3/AOPEP* and *SUOX* showed increased expression during prepuberty in testis compared to fetal life (**Figure S3**). However, this expression declined to very low levels at puberty and in adulthood. In the metabolic tissues, *C9orf3/AOPEP* and *SUOX* expression increased significantly after birth (**Figures S3A, B**). *C9orf3/AOPEP* expression increase slightly in the brain tissues towards adulthood (**Figure S3A**). *SUMO1P1* expression was undetectable until puberty and then increased dramatically towards adulthood in testis, whereas no expression was detected in any other postnatal tissues (**Figure S3C**).

Cell surface receptor genes (*ERBB3, ERBB4, PLGRKT*) were dynamically expressed postnatally (**Figure S4**). *ERBB3* expression levels remained low and constant in the heart, kidney and brain tissues while levels increased with age in the liver and kidney. In the testis, *ERBB3* expression was high during prepuberty, but levels declined significantly at puberty where it remained low during late puberty and adult life (**Figure S4A**). *ERBB4* expression was least in the liver and testis tissues, but levels remained constant in the brain. Compared to fetal life, *ERBB4* expression decreased during prepuberty in heart and kidney samples but levels remained low and constant thereafter in the heart (**Figure S4B**). *PLGRKT* was highest expressed in heart tissues postnatally and the expression increased with age. Levels also increased slightly from birth till prepuberty in kidney samples whilst expression remained relatively constant in testis, liver and brain tissues (**Figure S4C**). Postnatally *FBN3* was not expressed in any tissues (**Figure S5**).

Metabolic genes (*INSR, FDFT1*) were expressed dynamically from birth throughout lifetime in all tissues (**Figure S6**). In the testis, *INSR* levels decreased from birth until adulthood, while *FDFT1* levels increased. The expression of both genes remained constant during prepuberty, puberty and adulthood. The levels of *INSR* expression in the liver appeared slightly higher postnatally compared to fetal life, whereas those in kidney and heart were relatively unchanged. Relative to fetal expression, *FDFT1* declined towards puberty/adulthood more in the liver, and only slightly in the brain tissues.

The expression of all reproductive genes (*FSHB, FSHR, LHCGR, AMH* and *AR*) was very low or nil at the postnatal stages for both brain tissues and the three metabolic tissues, except for high *FSHB* expression in prepubertal kidney and high *AR* expression in the liver (**Figure S7**). *AMH* level was also low or not detected in postnatal testes (**Figure S7E**). *FSHB* showed an increased expression in the testis during puberty followed by a decline during adulthood, whereas *FSHR* was low prepubertally, then increased expression at puberty and the levels remained high throughout adulthood (**Figures S7A, B**). Contrary to this, expression of *AR* in the testis had been increased towards mid-gestation, remained high during prepuberty, but then declined during puberty and adulthood (**Figure S7D**). *LHCGR* expression levels were slightly higher in some pubertal and adult testis samples than in others, but generally low (**Figure S7C**).

## Discussion

In this study, we analysed the expression patterns of genes in loci associated with PCOS (candidate genes) from 4-20 weeks of gestation as an index of their potential roles in the development of PCOS at different stages of postnatal human life in gonadal, metabolic and brain tissues. We found that candidate genes such as *HMGA2, TOX3* and *FBN3* were mainly expressed in fetal tissues, while *DENND1A, THADA, MAPRE1, RAB5B, ARL14EP, KRR1, NEIL2*, and *RAD50* were dynamically expressed in all postnatal tissues studied. Notably, the expression patterns of PCOS candidate genes observed in the human ovary were consistent to our previous findings in bovine ovaries at the same timeframe (17–19). However, due to the limitation of obtaining human fetal tissues after mid-gestation, it was not prudent to cluster or group the PCOS candidate genes into expressed early, late or throughout gestation as previously done for bovine fetal ovaries (17–19). We therefore grouped these genes based on their functionality, while we focussed on the differences and similarities in each group. Although, postnatal ovary samples were not available and postnatal kidney samples were only available up to 8 years of age, the findings of this study infer that other fetal tissues, in addition to the ovary, could also be involved in the manifestation of the syndrome in adulthood.

It is important to recognise that GWAS findings only account for a small fraction of the estimated heritability of PCOS and do not identify specific genes but rather loci related to the syndrome (22). Genes within or near these loci could either have a causal or regulatory role in PCOS, which needs to be investigated further (23). This may be addressed by Mendelian randomisation studies and transcriptome-wide association studies in the future. Numerous human and animal studies have focussed on defining the possible causal or regulatory roles of these genes. For instance, recent animal studies have associated *THADA* and *RAD50* with ovarian folliculogenesis, steroidogenesis, and female fertility (24–26). However, studies comparing PCOS adult tissues with controls rarely identify PCOS candidate genes to be differentially expressed (19, 27). A study comparing the ovaries of PCOS women with controls showed no significant difference in the expression of these candidate genes except for *RAD50* (19). A meta-analysis involving lean (BMI ≤ 23) and obese (BMI ≥ 23) PCOS patients identified *ZBTB16, FSHR, GATA4* and *AR* to be downregulated in cumulus cells of lean PCOS women, while *INSR, THADA, PLGRKT* were downregulated in endometrial tissues of obese women with PCOS (27). These findings not only suggest roles for the different organs other than the ovary in the pathophysiology of PCOS, but could also imply that most of these candidate genes could be dysregulated during early stages of fetal development when some are mostly expressed. Thus, further understanding of the effects of androgens, AMH, TGFβ and other regulatory factors (18) on fetal programming of candidate genes during development is required.

The expression patterns as well as the roles/mechanisms of these candidate genes during normal fetal development and postnatally could be of value in identifying abnormalities that could lead to PCOS in adulthood. Considering the limitation of collecting human fetal samples after mid-gestation and also the strong similarities between human and bovine ovaries in morphology and physiology, gestational length and the propensity for singleton pregnancies, implications can be drawn from bovine data. In addition, the expression level of a candidate gene in a particular tissue at birth could inform to some extent the level of expression for this gene during the final days of gestation. For instance, it can be inferred that *HMGA2* levels might be consistently very low during the third trimester in all human tissues as the levels at birth were the same as those at mid-gestation, consistent with previous bovine studies (17–19). More so, the correlation between candidate genes within a tissue during fetal development suggests a possible co-regulation between these genes, which needs to be further investigated. This has become necessary as some loci identified in GWAS contain up to three candidate genes in/near it, requiring more studies to understand their possible association with the syndrome. For instance, although the possible role of *FDFT1* in PCOS aetiology remains unknown, it is located in GWAS loci 8p32.1 in proximity to *GATA4* and *NEIL2*; it is not clear if all these genes are causal or regulatory in PCOS (28).

Correlation studies provide preliminary knowledge about the relationships between candidate genes, their possible co-regulation (either co-activation/-inhibition) and affirm that the genes do not necessarily operate in isolation. In this study, correlation of *HMGA2/YAP1* and *YAP1/RAD50* was significant in at least 5 out of the 7 tissues studied. *HMGA2* plays a crucial role in proliferation and differentiation of mesenchymal cells and is also involved in adipogenesis, stem cell development as well as spermatogenesis (29–32). It also increases the proliferation of cancer cells by promoting cell cycle entry and apoptosis inhibition (33). *HMGA2* has been associated with the Hippo-YAP pathway as it regulates *YAP1* stability and possibly inhibits its ubiquitination (34). Although these genes have been studied individually in PCOS, the co-expression of *HMGA2* and *YAP1* in PCOS has yet to be studied. *HMGA2* has been associated with polycystic ovary morphology (PCOM) phenotype in PCOS patients of Han Chinese ancestry, potentially functioning to promote the proliferation of ovarian granulosa cells *via* the HMGA2/IMP2 pathway, thereby underpinning the increased proliferation of early-growing follicles and decreased apoptosis in granulosa cells in PCOS (35–37). *HMGA2* has also been significantly associated with both hyperandrogenism and oligo/amenorrhea in women with PCOS in Saudi Arabia (38). More so, *YAP1* is highly expressed in mammalian oocytes and preimplantation embryos, consistent with our finding in 4 week fetal tissues (39). *YAP1* is a core component of the Hippo signalling pathway, essential for cell proliferation and apoptosis during early developmental events, promoting organ size and tumorigenesis (40–43). The gene is also important for normal ovarian development and function, and is required for proliferation of granulosa cells (44). Furthermore, maternal accumulation of *YAP1* in the oocyte is crucial for zygotic genome activation, which occurs 2-3 days after fertilisation (39). In ovarian granulosa cells in PCOS, a significant decrease in methylation level was observed in the promoter region of *YAP1*, accounting for a significant increase in the mRNA and protein expression levels of YAP1 (45). Treatment of granulosa cells from control women with testosterone, but not luteinizing hormone (LH) or follicle stimulating hormone (FSH) reduced *YAP1* methylation in a dose-dependent manner (45); implying regulatory roles of androgens on the gene. Together, these findings and the co-regulatory effects of *HMGA2* and *YAP1* identified in this study, require further exploration towards delineating their roles in the pathogenesis of PCOS.

Although the cause of PCOS remains unclear, it is well known that aberration in most hormones involved in folliculogenesis and ovarian steroidogenesis interfere with the feedback mechanisms that regulate both processes. Hormones such as LH, insulin, AMH and androgens are elevated whilst FSH levels are reduced. This, in addition to hyperandrogenism in most women with PCOS, disrupted hypothalamo-pituitary function leading to increased LH pulse frequency, increased LH/FSH ratio and a persistently rapid frequency of gonadotropin-releasing hormone (GnRH) pulse secretion, suggests an impaired feedback mechanism between gonads and the brain (46). The anomalous levels of these hormones further cause abnormal oocyte maturation and premature luteinisation of granulosa cells leading to premature arrest of activated follicles at the antral stage. This then results in the accumulation of small to medium antral follicles in polycystic ovaries as well as excess androgen production (47). In this study we observed, as expected, that mRNA of reproductive genes (*FSHB*, *FSHR*, *LHCGR*, *AR*, *AMH*) were not detected during the first half of fetal development in the tissues studied except for *LHCGR* and *AMH* in the testis. These genes are usually expressed during the second half of gestation in the ovary and are associated with folliculogenesis and steroidogenesis (17, 18); the lack of *AMH* in the ovary in first half of gestation is consistent with its known function in regulating Mullerian tract regression and the lack of *AMH* and *LHCGR* with the absence of follicles at early stages of ovary development (48).

Furthermore, the increased risk of metabolic disorders such as obesity, chronic hypertension and pre-gestational diabetes in pregnant women with PCOS (49) implies that dysregulation of candidate genes in metabolic tissues could certainly play a role in the aetiology of the syndrome. Thus, insulin receptor signalling has also been associated with GnRH dysregulation leading to LH secretion and reproductive dysfunction in obesity (50). Insulin resistance has also been linked with increased androgen levels among PCOS patients. Specifically, both overexpression of insulin receptor (INSR) in the ovaries of non-obese PCOS patients and its underexpression in metabolic tissues of obese PCOS patients results in feedback mechanisms for excess ovarian androgen production (22). Moreover, offspring of women with PCOS are more likely to have metabolic and congenital anomalies compared to those from healthy women (49, 51, 52). Notably, brothers/sons of women with PCOS have elevated androgen levels (53), increased total cholesterol and low density lipoproteins levels at puberty (16), decreased insulin sensitivity (independent of obesity) and glucose tolerance (12), among other symptoms. Also, hepatic dysfunctions and risk of liver diseases have been observed in PCOS models in male sheep (11), female sheep (54, 55), and rats (56, 57), together affirming the roles of these organs in the pathophysiology of the syndrome. Although transcriptional and post-transcriptional factors were not evaluated, *INSR* and *FDFT1* were dynamically expressed in all tissues examined in this study. However, it is not clear how dysregulation of these and other genes during fetal development are involved in the metabolic symptoms observed in PCOS. Thus, understanding the roles of PCOS candidate genes in these metabolic tissues and their possible dysregulation in PCOS will improve understanding of the pathogenesis of disorder.

Association studies as well as co-localisation studies have been carried out on genes in loci genetically associated with PCOS with the hope to map the role of these candidate genes to the phenotypes or symptoms observed in women with PCOS [reviewed in (38, 58–67)]. Specifically, *FSHB* and *FHSR* loci have been associated with gonadotropin levels, while *LHCGR, FSHR, DENND1A, RAB5/SUOX, HMGA2, C9orf3, YAP1, TOX3, RAD50, FBN3*, and *AMH* have been associated with gonadotropin action and ovarian function (61, 62, 64). *THADA, GATA/NEIL2, ERBB4, SUMO1P1, INSR, KRR1* and *RAB5B* have been associated with metabolic function (61, 62, 64, 65). However, most of these studies, including GWAS, were carried out in adult women with confirmed diagnosis, mostly several years after presenting their first symptoms. Considering the increasing evidence on the fetal origin of PCOS, studies monitoring high-risk children from birth until adulthood where phenotypes are observed, should be the focus of current studies for delineating this polygenic disorder. This supports the recommendations of the International Guidelines which have emphasised the metabolic nature of PCOS (68).

It is tempting to infer that dysregulation of genes expressed in particular tissues could have relevant functions or mechanisms contributing to the predisposition of the syndrome in these tissues. The metabolic abnormalities observed particularly in male offspring or brothers of women with PCOS also present empirical evidence that PCOS is not only a syndrome of the ovary. Collectively, the role of candidate genes in various tissues, if clearly defined, could inform/guide further studies into delineating the possible mechanisms that are involved in PCOS predisposition in different tissues from conception till adulthood.

## Conclusions

Although, Mendelian randomisation studies and transcriptome-wide association studies were not included in this study, they may be addressed in future studies. Also, the influence of transcriptional and post-transcriptional mechanisms such as mRNA stability/degradation, storage in stress granules, translational control on gene expression should be considered. That notwithstanding, this study further confirms that PCOS is a polygenic syndrome involving multiple organs of the body. This study identified expression of PCOS candidate genes during fetal development of many organs in humans. Thus, the fetal origin of a predisposition to PCOS in adulthood could arise *via* the effects of PCOS candidate genes in the development of multiple organs.

## Data availability statement

The datasets presented in this study can be found in online repositories. The names of the repository/repositories and accession number(s) can be found in the article/**Supplementary Material**.

## Author contributions

RA, KH and RR designed the study. RA performed statistical analysis. RA, KH and RR interpreted the data and contributed to discussion. RA, KH, RAA and RR wrote the manuscript. RR is the guarantor of this work, had full access to all the data in the study, and assumes full responsibility for the integrity of the data and the accuracy of the data analysis. All authors contributed to the article and approved the submitted version.
