# Supplementary material for: Genes in loci genetically associated with polycystic ovary syndrome are dynamically expressed in human fetal gonadal, metabolic and brain tissues
Source: Front Endocrinol (Lausanne). 2023 May 8;14:1149473. doi: 10.3389/fendo.2023.1149473 (PMC10201802; doi:10.3389/fendo.2023.1149473)
Supplement: Supplementary file 2 [file Table_2.pdf]

Table S4

Pearson's correlation coefficients (R) between mRNA expression levels of *GATA4* and PCOS candidate genes in fetal organs and the number of organs with significant ($P < 0.01$) correlations.

Genes	Ovary (N=18)	Testis (N=27)	Heart (N=38)	Kidney (N=29)	Liver (N=37)	Cerebellum (N=33)	Brain (N=32)	Number of organs[†]
age (days)	0.45	-0.15	-0.51[b]	-0.53[b]	-0.66[d]	-0.03	-0.22	3-
FSHB	-	-	-	-0.14	-	-0.11	-	
FSHR	0.79[d]	0.63[c]	0.20	0.59[b]	0.66[d]	-0.14	0.03	4+
LHCGR	-0.64[b]	0.66[c]	-0.19	0.05	0.42[a]	-0.19	0.05	1+, 1-
AR	0.25	-0.49[a]	-0.24	0.39[a]	-0.17	-0.12	0.02	
AMH	0.34	0.72[d]	0.09	0.18	0.11	-0.06	0.34	1+
INSR	0.35	0.73[d]	0.24	0.01	-0.68[d]	0.23	-0.17	1+, 1-
FDFT1	-0.18	0.65[c]	0.04	0.38[a]	0.24	0.06	-0.02	1+
ERBB3	0.15	0.76[d]	0.07	-0.48[a]	0.18	-0.09	-0.10	1+
ERBB4	-0.77[c]	-0.20	0.18	-0.43[a]	0.63[d]	0.21	0.02	1+, 1-
PLGRKT	-0.41	0.45[a]	-0.42[a]	-0.28	-0.24	-0.14	0.07	
HMGA2	-0.82[d]	-0.25	0.33	0.70[d]	0.94[d]	-0.14	-0.08	2+, 1-
TOX3	0.56[a]	-0.23	-0.10	-0.35	0.59[c]	0.32	0.12	1+
GATA4	*	*	*	*	*	*	*	
YAP1	-0.77[c]	-0.60[b]	-0.08	-0.04	0.86[d]	-0.10	-0.05	1+, 2-
ZBTB16	0.13	-0.47[a]	0.16	-0.07	0.11	-0.02	-0.05	
IRF1	0.57[a]	0.02	0.28	-0.33	-0.50[b]	0.09	-0.18	1-
NEIL2	0.61[b]	0.62[c]	0.57[c]	0.71[d]	-0.32	0.08	-0.22	4+
RAD50	0.03	-0.09	0.01	0.24	0.30	0.11	-0.18	
KRR1	0.07	-0.22	-0.33	-0.18	-0.26	0.23	-0.11	
RAB5B	-0.43	0.06	0.00	-0.17	0.14	0.20	-0.23	
ARL14EP	-0.40	-0.24	-0.58[c]	0.00	0.28	0.29	-0.19	1-
DENND1A	0.76[c]	0.35	0.60[c]	-0.01	0.19	0.21	-0.23	2+
THADA	0.17	0.14	0.11	-0.11	0.45[b]	-0.04	-0.13	1+
MAPRE1	-0.02	0.56[b]	0.22	0.35	0.11	0.19	-0.03	1+
AOPEP	0.15	0.10	-0.08	-0.56[b]	-0.18	-0.27	-0.01	1-
SUOX	0.04	0.07	-0.08	-0.20	0.12	-0.21	0.01	
SUMO1P1	0.66[b]	0.09	-0.11	0.15	-0.10	-	0.23	1+
FBN3	0.30	0.09	0.39[a]	0.25	0.86[d]	-0.20	-0.09	1+

Positive and negative correlations are marked in pink and blue, respectively. The colour intensity corresponds with the strength of the correlation. *P*-values: a < 0.05; b < 0.01; c < 0.001; d < 0.0001. Tissues with $P < 0.01$ were regarded as significant. [†]Number of organs with significant ($P < 0.01$) positive (+) or negative (-) correlations. '-' and '*' indicate no gene expression and correlation between same gene, respectively.

Table S5

Pearson's correlation coefficients (R) between mRNA expression levels of *TOX3* and PCOS candidate genes in fetal organs and the number of organs with significant ($P < 0.01$) correlations.

Genes	Ovary (N=18)	Testis (N=27)	Heart (N=38)	Kidney (N=29)	Liver (N=37)	Cerebellum (N=33)	Brain (N=32)	Number of organs[‡]
age (days)	0.34	-0.50[b]	-0.31	0.68[d]	-0.51[b]	-0.60[c]	-0.07	1+, 3-
FSHB	-	-	-	0.45[a]	-	-0.22	-	
FSHR	0.47[a]	-0.21	0.37[a]	-0.31	0.34[a]	-0.17	0.08	
LHCGR	-0.27	-0.45[a]	0.06	-0.30	0.54[c]	-0.22	0.01	1+
AR	0.28	-0.25	-0.08	-0.08	-0.13	0.15	0.36[a]	
AMH	-0.13	-0.70[d]	-0.05	-0.60[c]	-0.19	-0.04	0.08	2-
INSR	0.50[a]	-0.55[b]	-0.26	0.62[c]	-0.24	0.33	-0.10	1+, 1-
FDFT1	0.27	-0.41[a]	0.81[d]	-0.38[a]	0.31	0.72[d]	0.05	2+
ERBB3	0.16	-0.36	0.55[c]	0.51[b]	0.07	-0.14	-0.14	2+
ERBB4	-0.35	0.56[b]	-0.23	0.63[c]	0.32	0.33	0.65[d]	3+
PLGRKT	-0.47[a]	-0.29	-0.26	0.44[a]	-0.17	0.44[b]	-0.30	1+
HMGA2	-0.59[a]	0.86[d]	0.58[c]	-0.54[b]	0.62[d]	0.11	0.05	3+, 1-
TOX3	*	*	*	*	*	*	*	
GATA4	0.56[a]	-0.23	-0.10	-0.35	0.59[c]	0.32	0.12	1+
YAP1	-0.31	0.63[c]	0.04	0.31	0.67[d]	0.25	0.16	2+
ZBTB16	0.55[a]	-0.13	-0.06	0.45[a]	0.28	0.20	0.09	
IRF1	0.47[a]	-0.47[a]	-0.11	0.52[b]	-0.44[b]	-0.66[d]	-0.25	1+, 2-
NEIL2	0.69[b]	-0.62[c]	0.05	-0.49[a]	-0.28	-0.58[c]	0.05	1+, 2-
RAD50	0.55[a]	0.53[b]	0.21	0.16	0.37[a]	0.50[b]	0.21	2+
KRR1	0.58[a]	0.32	0.00	0.35	0.12	0.58[c]	0.43[a]	1+
RAB5B	-0.06	0.26	0.01	0.56[b]	0.23	0.18	0.45[b]	2+
ARL14EP	0.19	0.17	-0.18	0.33	0.60[d]	0.49[b]	0.21	2+
DENND1A	0.63[b]	0.15	0.45[b]	0.09	-0.06	-0.43[a]	0.13	2+
THADA	0.76[c]	0.35	0.40[a]	0.38	0.26	0.50[b]	0.10	2+
MAPRE1	0.56[a]	0.06	0.06	-0.34	0.20	0.77[d]	0.23	1+
AOPEP	-0.09	-0.28	-0.32	0.60[b]	-0.16	-0.08	0.02	1+
SUOX	-0.20	0.03	0.12	0.23	-0.09	-0.25	0.45[b]	1+
SUMO1P1	0.27	-0.08	0.29	-0.13	0.03	-	-0.21	
FBN3	-0.05	0.62[c]	0.52[b]	-0.58[b]	0.56[c]	-0.51[b]	0.02	3+, 2-

Positive and negative correlations are marked in pink and blue, respectively. The colour intensity corresponds with the strength of the correlation. *P*-values: a < 0.05; b < 0.01; c < 0.001; d < 0.0001. Tissues with $P < 0.01$ were regarded as significant. [‡]Number of organs with significant ($P < 0.01$) positive (+) or negative (-) correlations. '-' and '*' indicate no gene expression and correlation between same gene, respectively.

Table S6

Pearson's correlation coefficients (R) between mRNA expression levels of *ZBTB16* and PCOS candidate genes in fetal organs and the number of organs with significant ($P < 0.01$) correlations.

Genes	Ovary (N=18)	Testis (N=27)	Heart (N=38)	Kidney (N=29)	Liver (N=37)	Cerebellum (N=33)	Brain (N=32)	Number of organs[†]
age (days)	0.43	0.62[c]	0.43[a]	0.25	0.15	-0.66[d]	-0.57[c]	1+, 2-
FSHB	-	-	-	0.58[b]	-	-0.12	-	1+
FSHR	0.15	-0.21	-0.17	-0.12	0.14	0.47[b]	0.77[d]	2+
LHCGR	0.03	-0.17	-0.25	-0.39[a]	0.04	0.53[b]	0.88[d]	2+
AR	0.32	0.52[b]	-0.22	-0.07	0.52[c]	0.80[d]	0.84[d]	4+
AMH	-0.52[a]	-0.35	-0.42[a]	-0.12	-0.38[a]	-0.38[a]	-0.19	
INSR	0.15	-0.02	0.55[c]	0.43[a]	0.16	-0.40[a]	-0.57[c]	1+, 1-
FDFT1	0.67[b]	-0.16	0.12	-0.19	0.05	0.40[a]	-0.18	1+
ERBB3	0.06	-0.36	-0.15	0.37	0.14	0.83[d]	0.88[d]	2+
ERBB4	-0.06	-0.07	0.36[a]	-0.01	0.14	-0.26	0.01	
PLGRKT	-0.54[a]	-0.55[b]	0.02	-0.07	-0.01	0.02	-0.38[a]	1-
HMGA2	-0.40	-0.26	-0.14	-0.27	0.20	0.86[d]	0.76[d]	2+
TOX3	0.55[a]	-0.13	-0.06	0.45[a]	0.28	0.20	0.09	
GATA4	0.13	-0.47[a]	0.16	-0.07	0.11	-0.02	-0.05	
YAP1	-0.10	0.40[a]	0.22	-0.42[a]	0.30	0.87[d]	0.91[d]	2+
ZBTB16	*	*	*	*	*	*	*	
IRF1	0.56[a]	0.37	0.39[a]	0.64[c]	0.02	-0.04	0.14	1+
NEIL2	0.70[b]	-0.03	-0.10	0.01	0.18	-0.31	-0.20	1+
RAD50	0.71[c]	0.09	0.31	-0.43[a]	0.70[d]	0.69[d]	0.56[c]	4+
KRR1	0.34	0.29	0.19	-0.46[a]	0.52[b]	-0.51[b]	0.50[b]	2+, 1-
RAB5B	0.43	0.34	0.52[b]	0.53[b]	0.70[d]	0.19	0.11	3+
ARL14EP	-0.02	0.08	0.09	-0.37	0.17	-0.13	-0.24	
DENND1A	0.35	0.05	0.41[a]	0.62[c]	0.22	-0.36[a]	0.04	1+
THADA	0.74[c]	-0.02	0.21	-0.10	0.39[a]	0.82[d]	0.79[d]	3+
MAPRE1	0.76[c]	-0.32	-0.17	-0.47[a]	0.59[c]	0.48[b]	0.38[a]	3+
AOPEP	-0.33	-0.07	0.11	0.47[a]	-0.48[b]	0.61[c]	0.91[d]	2+, 1-
SUOX	-0.05	-0.41[a]	-0.47[b]	0.66[c]	-0.26	-0.03	0.32	1+, 1-
SUMO1P1	-0.06	0.06	-0.26	-0.13	-0.22	-	-0.20	
FBN3	-0.50[a]	-0.36	-0.33	-0.07	0.18	0.23	0.24	

Positive and negative correlations are marked in pink and blue, respectively. The colour intensity corresponds with the strength of the correlation. *P*-values: a < 0.05; b < 0.01; c < 0.001; d < 0.0001. Tissues with $P < 0.01$ were regarded as significant. [†]Number of organs with significant ($P < 0.01$) positive (+) or negative (-) correlations. '-' and '*' indicate no gene expression and correlation between same gene, respectively.

Table S7

Pearson's correlation coefficients (R) between mRNA expression levels of *IRF1* and PCOS candidate genes in fetal organs and the number of organs with significant ($P < 0.01$) correlations.

Genes	Ovary (N=18)	Testis (N=27)	Heart (N=38)	Kidney (N=29)	Liver (N=37)	Cerebellum (N=33)	Brain (N=32)	Number of organs[†]
age (days)	0.69[b]	0.84[d]	0.11	0.67[c]	0.82[d]	0.54[b]	0.04	5+
FSHB	-	-	-	0.60[c]	-	0.01	-	1+
FSHR	0.39	0.42[a]	0.18	-0.30	-0.21	0.20	0.11	
LHCGR	-0.53[a]	0.48[a]	-0.01	-0.28	-0.24	0.29	0.07	
AR	0.52[a]	0.67[c]	-0.01	0.10	0.52[c]	0.10	0.17	2+
AMH	-0.25	0.09	-0.31	-0.32	-0.29	-0.34	-0.23	
INSR	0.08	0.42[a]	0.21	0.42[a]	0.46[b]	-0.21	-0.14	1+
FDFT1	0.32	0.51[b]	0.24	-0.41[a]	-0.27	-0.59[c]	-0.35[a]	1+, 1-
ERBB3	0.01	0.14	0.20	0.48[a]	0.06	0.26	0.13	
ERBB4	-0.60[b]	-0.44[a]	0.11	0.32	-0.23	-0.25	0.08	1-
PLGRKT	-0.59[a]	-0.29	-0.11	0.17	0.22	-0.49[b]	-0.04	1-
HMGA2	-0.62[b]	-0.72[d]	0.19	-0.71[d]	-0.42[b]	0.02	0.25	3-
TOX3	0.47[a]	-0.47[a]	-0.11	0.52[b]	-0.44[b]	-0.66[d]	-0.25	2+, 1-
GATA4	0.57[a]	0.02	0.28	-0.33	-0.50[b]	0.09	-0.18	1-
YAP1	-0.56[a]	-0.16	0.14	-0.42[a]	-0.36[a]	-0.05	0.17	
ZBTB16	0.56[a]	0.37	0.39[a]	0.64[c]	0.02	-0.04	0.14	1+
IRF1	*	*	*	*	*	*	*	
NEIL2	0.83[d]	0.55[b]	0.59[c]	-0.16	0.23	0.59[c]	0.13	4+
RAD50	0.41	0.14	0.17	-0.51[b]	-0.02	-0.14	-0.04	1-
KRR1	-0.02	0.08	0.06	-0.31	0.12	-0.21	0.03	
RAB5B	0.21	0.48[a]	0.27	0.65[c]	0.29	0.21	0.09	1+
ARL14EP	-0.33	0.12	-0.06	-0.23	-0.02	-0.17	-0.40[a]	
DENND1A	0.49[a]	-0.02	0.34	0.44[a]	-0.12	0.51[b]	0.07	1+
THADA	0.44	-0.09	0.15	-0.20	-0.29	-0.25	0.10	
MAPRE1	0.39	0.18	0.02	-0.64[c]	0.21	-0.50[b]	-0.13	2-
AOPEP	-0.02	-0.19	-0.10	0.62[c]	-0.19	0.19	0.14	1+
SUOX	0.21	-0.34	-0.33	0.49[b]	-0.30	0.27	0.11	1+
SUMO1P1	0.11	0.06	-0.11	0.26	0.14	-	0.27	
FBN3	-0.35	-0.75[d]	0.09	-0.51[b]	-0.38[a]	0.42[a]	-0.02	2-

Positive and negative correlations are marked in pink and blue, respectively. The colour intensity corresponds with the strength of the correlation. *P*-values: a < 0.05; b < 0.01; c < 0.001; d < 0.0001. Tissues with $P < 0.01$ were regarded as significant. [†]Number of organs with significant ($P < 0.01$) positive (+) or negative (-) correlations. '-' and '*' indicate no gene expression and correlation between same gene, respectively.

Table S8

Pearson's correlation coefficients (R) between mRNA expression levels of *NEIL2* and PCOS candidate genes in fetal organs and the number of organs with significant ($P < 0.01$) correlations.

Genes	Ovary (N=18)	Testis (N=27)	Heart (N=38)	Kidney (N=29)	Liver (N=37)	Cerebellum (N=33)	Brain (N=32)	Number of organs[†]
age (days)	0.75[c]	0.31	-0.42[a]	-0.55[b]	0.27	0.75[d]	0.67[d]	3+, 1-
FSHB	-	-	-	0.04	-	0.28	-	-
FSHR	0.36	0.63[c]	0.58[c]	0.41[a]	0.05	-0.09	-0.12	2+
LHCGR	-0.34	0.73[d]	0.30	0.26	-0.10	-0.19	-0.20	1+
AR	0.31	-0.11	0.28	0.39[a]	0.20	-0.22	-0.33	
AMH	-0.24	0.63[c]	-0.08	0.56[b]	-0.31	-0.07	-0.23	2+
INSR	0.19	0.71[d]	0.00	0.06	0.28	-0.22	0.22	1+
FDFT1	0.47[a]	0.76[d]	0.37[a]	0.49[b]	0.16	-0.45[b]	-0.18	2+, 1-
ERBB3	-0.13	0.62[c]	0.56[c]	-0.60[c]	-0.39[a]	-0.06	-0.05	2+, 1-
ERBB4	-0.55[a]	-0.39[a]	0.00	-0.76[d]	0.01	0.03	0.26	1-
PLGRKT	-0.71[c]	0.20	-0.43[a]	-0.51[b]	0.47[b]	-0.40[a]	-0.25	1+, 2-
HMGA2	-0.78[c]	-0.60[b]	0.53[b]	0.67[c]	-0.17	-0.30	-0.34	2+, 2-
TOX3	0.69[b]	-0.62[c]	0.05	-0.49[a]	-0.28	-0.58[c]	0.05	1+, 2-
GATA4	0.61[b]	0.62[c]	0.57[c]	0.71[d]	-0.32	0.08	-0.22	4+
YAP1	-0.61[b]	-0.63[c]	0.13	-0.42[a]	-0.14	-0.35[a]	-0.35	2+
ZBTB16	0.70[b]	-0.03	-0.10	0.01	0.18	-0.31	-0.20	1-
IRF1	0.83[d]	0.55[b]	0.59[c]	-0.16	0.23	0.59[c]	0.13	4-
NEIL2	*	*	*	*	*	*	*	
RAD50	0.48[a]	-0.23	0.18	-0.18	0.01	-0.50[b]	-0.29	1-
KRR1	0.26	-0.36	-0.07	-0.49[b]	0.31	-0.54[b]	-0.21	2-
RAB5B	-0.01	0.24	0.09	-0.18	0.02	0.18	0.12	
ARL14EP	-0.32	-0.47[a]	-0.29	-0.33	-0.16	-0.20	0.22	
DENND1A	0.51[a]	0.25	0.52[b]	0.13	0.38[a]	0.68[d]	0.12	2+
THADA	0.56[a]	-0.04	0.33	-0.31	0.16	-0.49[b]	-0.48[b]	2-
MAPRE1	0.50[a]	0.48[a]	0.39[a]	0.32	0.36[a]	-0.48[b]	-0.50[b]	2-
AOPEP	-0.05	-0.08	-0.28	-0.41[a]	0.07	-0.26	-0.20	
SUOX	-0.08	-0.10	-0.10	-0.04	-0.49[b]	0.22	0.35	1-
SUMO1P1	0.21	0.02	0.11	0.10	0.03	-	0.25	
FBN3	-0.33	-0.43[a]	0.52[b]	0.52[b]	-0.12	0.18	0.27	2+

Positive and negative correlations are marked in pink and blue, respectively. The colour intensity corresponds with the strength of the correlation. *P*-values: a < 0.05; b < 0.01; c < 0.001; d < 0.0001. Tissues with $P < 0.01$ were regarded as significant. [†]Number of organs with significant ($P < 0.01$) positive (+) or negative (-) correlations. '-' and '*' indicate no gene expression and correlation between same gene, respectively.

Table S9

Pearson's correlation coefficients (R) between mRNA expression levels of *KRR1* and PCOS candidate genes in fetal organs and the number of organs with significant ($P < 0.01$) correlations.

Genes	Ovary (N=18)	Testis (N=27)	Heart (N=38)	Kidney (N=29)	Liver (N=37)	Cerebellum (N=33)	Brain (N=32)	Number of organs[†]
age (days)	0.01	0.29	0.34	0.30	0.36[a]	-0.58[c]	-0.28	1-
FSHB	-	-	-	-0.04	-	-0.18	-	
FSHR	-0.06	0.06	0.17	-0.06	0.04	0.38[a]	0.27	
LHCGR	0.23	-0.21	0.19	0.20	-0.19	0.28	0.37[a]	
AR	0.16	0.39	0.40[a]	0.16	0.41[a]	0.56[c]	0.56[c]	1+, 1-
AMH	-0.33	-0.48[a]	-0.53[b]	-0.42[a]	-0.43[b]	-0.53[b]	-0.27	3-
INSR	0.51[a]	-0.05	0.24	-0.02	0.51[b]	0.32	-0.19	1+
FDFT1	0.14	-0.31	0.18	-0.17	-0.08	0.36[a]	-0.14	
ERBB3	0.19	-0.05	0.18	-0.02	-0.33[a]	0.26	0.38[a]	
ERBB4	0.15	0.18	0.63[d]	0.54[b]	0.04	0.38[a]	0.39[a]	4+
PLGRKT	-0.20	-0.25	0.24	0.53[b]	-0.06	0.14	-0.30	1+
HMGA2	-0.12	0.09	-0.05	-0.09	-0.08	0.54[b]	0.50[b]	2+
TOX3	0.58[a]	0.32	0.00	0.35	0.12	0.58[c]	0.43[a]	1+
GATA4	0.07	-0.22	-0.33	-0.18	-0.26	0.23	-0.11	
YAP1	0.23	0.61[c]	0.86[d]	0.91[d]	0.07	0.65[d]	0.57[c]	5+
ZBTB16	0.34	0.29	0.19	-0.46[a]	0.52[b]	0.51[b]	0.50[b]	3+
IRF1	-0.02	0.08	0.06	-0.31	0.12	-0.21	0.03	
NEIL2	0.26	-0.36	-0.07	-0.49[b]	0.31	-0.54[b]	-0.21	2-
RAD50	0.52[a]	0.63[c]	0.71[d]	0.85[d]	0.44[b]	0.90[d]	0.70[d]	6+
KRR1	*	*	*	*	*	*	*	
RAB5B	-0.08	0.34	0.51[b]	-0.20	0.44[b]	0.44[a]	0.45[a]	2+
ARL14EP	0.56[a]	0.73[d]	0.87[d]	0.90[d]	0.28	0.65[d]	0.32	4+
DENND1A	0.07	-0.13	-0.06	-0.65[c]	-0.04	-0.47[b]	-0.11	2-
THADA	0.48[a]	0.30	0.56[c]	0.52[b]	0.06	0.75[d]	0.58[c]	4+
MAPRE1	0.53[a]	0.06	0.39[a]	0.27	0.58[c]	0.70[d]	0.50[b]	3+
AOPEP	-0.08	-0.08	-0.20	-0.05	-0.12	0.25	0.44[a]	
SUOX	-0.22	-0.46[a]	-0.50[b]	-0.49[b]	-0.50[b]	-0.48[b]	0.09	4+
SUMO1P1	0.23	0.29	0.42[a]	0.03	-0.22	-	-0.10	
FBN3	-0.10	-0.12	-0.17	-0.39[a]	-0.02	-0.22	-0.01	

Positive and negative correlations are marked in pink and blue, respectively. The colour intensity corresponds with the strength of the correlation. *P*-values: a < 0.05; b < 0.01; c < 0.001; d < 0.0001. Tissues with $P < 0.01$ were regarded as significant. [†]Number of organs with significant ($P < 0.01$) positive (+) or negative (-) correlations. '-' and '*' indicate no gene expression and correlation between same gene, respectively.

Table S10

Pearson's correlation coefficients (R) between mRNA expression levels of *RAB5B* and PCOS candidate genes in fetal organs and the number of organs with significant ($P < 0.01$) correlations.

Genes	Ovary (N=18)	Testis (N=27)	Heart (N=38)	Kidney (N=29)	Liver (N=37)	Cerebellum (N=33)	Brain (N=32)	Number of organs[†]
age (days)	-0.04	0.27	0.33	0.54[b]	0.32	0.06	0.25	1+
FSHB	-	-	-	0.27	-	-0.15	-	
FSHR	-0.17	0.48[a]	0.39[a]	-0.11	0.19	0.09	0.05	
LHCGR	0.29	0.33	0.19	-0.46[a]	0.03	0.01	0.02	
AR	0.29	0.20	0.36[a]	0.07	0.49[b]	0.26	0.09	1+
AMH	-0.76[c]	-0.34	-0.71[d]	-0.54[b]	-0.58[c]	-0.76[d]	-0.51[b]	6-
INSR	-0.39	0.17	0.13	0.36	0.01	0.26	0.40[a]	
FDFT1	0.55[a]	0.40[a]	0.29	-0.14	0.20	-0.05	-0.22	
ERBB3	0.43	0.08	0.33	0.63[c]	0.25	0.08	0.05	1+
ERBB4	0.51[a]	0.27	0.53[b]	0.41[a]	0.18	0.54[b]	0.51[b]	3+
PLGRKT	0.05	-0.59[b]	-0.11	0.23	-0.07	-0.47[b]	-0.66[d]	3-
HMGA2	0.33	-0.05	0.12	-0.44[a]	0.24	0.11	-0.03	
TOX3	-0.06	0.26	0.01	0.56[b]	0.23	0.18	0.45[b]	2+
GATA4	-0.43	0.06	0.00	-0.17	0.14	0.20	-0.23	
YAP1	0.50[a]	0.40[a]	0.64[d]	-0.12	0.36[a]	0.18	0.06	1+
ZBTB16	0.43	0.34	0.52[b]	0.53[b]	0.70[d]	0.19	0.11	3+
IRF1	0.21	0.48[a]	0.27	0.65[c]	0.29	0.21	0.09	1+
NEIL2	-0.01	0.24	0.09	-0.18	0.02	0.18	0.12	
RAD50	0.59[a]	0.77[d]	0.80[d]	-0.22	0.86[d]	0.54[b]	0.49[b]	5+
KRR1	-0.08	0.34	0.51[b]	-0.20	0.44[b]	0.44[a]	0.45[a]	2+
RAB5B	*	*	*	*	*	*	*	
ARL14EP	0.24	0.10	0.18	-0.24	0.26	0.48[b]	0.30	1+
DENND1A	-0.22	0.27	0.43[a]	0.51[b]	0.32	0.11	0.33	1+
THADA	0.49[a]	0.41[a]	0.70[d]	0.24	0.39[a]	0.25	0.04	1+
MAPRE1	0.54[a]	0.64[c]	0.38[a]	-0.34	0.78[d]	0.27	0.23	2+
AOPEP	-0.66[b]	-0.71[d]	-0.50[b]	0.48[a]	-0.82[d]	-0.11	-0.01	4-
SUOX	0.22	-0.40[a]	-0.47[b]	0.38	-0.39[a]	-0.24	0.05	1-
SUMO1P1	-0.52[a]	0.15	0.13	0.17	-0.12	-	-0.23	
FBN3	-0.71[b]	-0.30	-0.11	-0.43[a]	0.16	-0.36[a]	0.23	1-

Positive and negative correlations are marked in pink and blue, respectively. The colour intensity corresponds with the strength of the correlation. *P*-values: a < 0.05; b < 0.01; c < 0.001; d < 0.0001. Tissues with $P < 0.01$ were regarded as significant. [†]Number of organs with significant ($P < 0.01$) positive (+) or negative (-) correlations. '-' and '*' indicate no gene expression and correlation between same gene, respectively.

Table S11

Pearson's correlation coefficients (R) between mRNA expression levels of *ARL14EP* and PCOS candidate genes in fetal organs and the number of organs with significant ($P < 0.01$) correlations.

Genes	Ovary (N=18)	Testis (N=27)	Heart (N=38)	Kidney (N=29)	Liver (N=37)	Cerebellum (N=33)	Brain (N=32)	Number of organs[†]
age (days)	-0.39	0.30	0.53[b]	0.32	-0.03	-0.02	0.30	1+
FSHB	-	-	-	-0.01	-	-0.03	-	
FSHR	-0.21	-0.13	-0.16	-0.04	0.32	-0.05	-0.26	
LHCGR	0.45	-0.20	0.13	0.35	0.42[a]	-0.15	-0.28	
AR	0.18	0.53[b]	0.27	0.31	0.15	-0.06	-0.21	1+
AMH	-0.23	-0.38	-0.31	-0.40[a]	-0.15	-0.37[a]	-0.33	
INSR	0.18	-0.13	0.27	0.14	-0.08	0.74[d]	0.53[b]	2+
FDFT1	-0.01	-0.31	-0.05	-0.24	0.13	0.12	0.21	
ERBB3	0.27	-0.02	-0.12	-0.07	-0.01	-0.25	-0.20	
ERBB4	0.55[a]	0.03	0.37[a]	0.52[b]	0.33[a]	0.74[d]	0.04	2+
PLGRKT	0.30	-0.05	0.55[c]	0.54[b]	0.06	0.09	-0.15	2
HMGA2	0.48[a]	-0.01	-0.34	-0.11	0.35[a]	-0.11	-0.18	
TOX3	0.19	0.17	-0.18	0.33	0.60[d]	0.49[b]	0.21	2+
GATA4	-0.40	-0.24	-0.58[c]	0.00	0.28	0.29	-0.19	1-
YAP1	0.72[c]	0.50[a]	0.56[c]	0.84[d]	0.47[b]	0.00	-0.20	4+
ZBTB16	-0.02	0.08	0.09	-0.37	0.17	-0.13	-0.24	
IRF1	-0.33	0.12	-0.06	-0.23	-0.02	-0.17	-0.40[a]	
NEIL2	-0.32	-0.47[a]	-0.29	-0.33	-0.16	-0.20	0.22	
RAD50	0.36	0.53[b]	0.30	0.83[d]	0.37[a]	0.47[b]	0.41[a]	3+
KRR1	0.56[a]	0.73[d]	0.82[d]	0.90[d]	0.28	0.65[d]	0.32	4+
RAB5B	0.24	0.10	0.18	-0.24	0.26	0.48[b]	0.30	1+
ARL14EP	*	*	*	*	*	*	*	
DENND1A	-0.35	-0.45[a]	-0.39[a]	-0.70[d]	-0.26	-0.22	0.03	1-
THADA	0.29	0.08	0.11	0.39[a]	0.19	0.19	-0.03	
MAPRE1	0.32	-0.07	0.10	0.25	0.21	0.43[a]	0.13	
AOPEP	0.04	0.02	0.18	-0.15	-0.17	-0.33	-0.24	
SUOX	-0.26	-0.25	-0.35[a]	-0.53[b]	-0.11	-0.47[b]	-0.22	2-
SUMO1P1	-0.35	0.06	0.28	0.01	0.22	-	-0.09	
FBN3	-0.10	-0.18	-0.44[b]	-0.43[a]	0.33[a]	-0.45[b]	0.16	2-

Positive and negative correlations are marked in pink and blue, respectively. The colour intensity corresponds with the strength of the correlation. *P*-values: a < 0.05; b < 0.01; c < 0.001; d < 0.0001. Tissues with $P < 0.01$ were regarded as significant. [†]Number of organs with significant ($P < 0.01$) positive (+) or negative (-) correlations. '-' and '*' indicate no gene expression and correlation between same gene, respectively.

Table S12

Pearson's correlation coefficients (R) between mRNA expression levels of *DENND1A* and PCOS candidate genes in fetal organs and the number of organs with significant ($P < 0.01$) correlations.

Genes	Ovary (N=18)	Testis (N=27)	Heart (N=38)	Kidney (N=29)	Liver (N=37)	Cerebellum (N=33)	Brain (N=32)	Number of organs[†]
age (days)	0.33	-0.14	-0.35[a]	0.01	-0.22	0.66[d]	0.09	1+
FSHB	-	-	-	0.14	-	0.07	-	
FSHR	0.68[b]	0.14	0.50[b]	-0.09	0.20	-0.34	-0.10	2+
LHCGR	-0.51[a]	0.24	-0.06	-0.49[b]	-0.08	-0.37[a]	0.00	1-
AR	0.19	-0.30	-0.12	-0.28	-0.29	-0.36[a]	-0.02	
AMH	0.24	0.15	-0.32	0.15	-0.22	0.02	-0.18	
INSR	0.49[a]	0.26	0.23	0.13	-0.15	0.07	0.28	
FDFT1	0.07	0.28	0.64[d]	0.09	0.33[a]	-0.62[d]	0.32	1+, 1-
ERBB3	0.12	-0.07	0.48[b]	0.34	-0.10	-0.19	0.01	1+
ERBB4	-0.58[a]	0.09	0.20	-0.20	0.13	0.01	-0.13	
PLGRKT	-0.36	-0.31	-0.47[b]	-0.23	0.05	-0.55[c]	-0.26	2-
HMGA2	-0.70[b]	0.09	0.62[c]	-0.05	0.18	-0.38[a]	0.01	1+, 1-
TOX3	0.63[b]	0.15	0.45[b]	0.09	-0.06	-0.43[a]	0.13	2+
GATA4	0.76[c]	0.35	0.60[c]	-0.01	0.19	0.21	-0.23	2+
YAP1	-0.60[b]	-0.04	0.18	-0.57[b]	0.18	-0.52[b]	0.00	2-
ZBTB16	0.35	0.05	0.41[a]	0.62[c]	0.22	-0.36[a]	0.04	1+
IRF1	0.49[a]	-0.02	0.34	0.44[a]	-0.12	0.51[b]	0.07	1+
NEIL2	0.51[a]	0.25	0.52[b]	0.13	0.38[a]	0.68[d]	0.12	2+
RAD50	0.03	0.11	0.44[b]	-0.62[c]	0.44[b]	-0.45[b]	0.03	2+, 2-
KRR1	0.07	-0.13	-0.06	-0.65[c]	-0.04	-0.47[b]	-0.11	2-
RAB5B	-0.22	0.27	0.43[a]	0.51[b]	0.32	0.11	0.33	1+
ARL14EP	-0.35	-0.45[a]	-0.39[a]	-0.70[d]	-0.26	-0.22	0.03	1-
DENND1A	*	*	*	*	*	*	*	
THADA	0.30	0.57[b]	0.55[c]	-0.10	0.58[c]	-0.56[c]	0.08	3+, 1-
MAPRE1	0.09	0.16	0.32	-0.33	0.55[c]	-0.55[c]	-0.02	1+, 1-
AOPEP	0.04	0.03	-0.31	0.45[a]	-0.46[b]	-0.26	-0.03	1-
SUOX	0.07	-0.10	-0.22	0.59[b]	-0.28	0.14	0.08	1+
SUMO1P1	0.54[a]	0.08	0.05	-0.06	-0.10	-	-0.29	
FBN3	0.24	0.18	0.47[b]	0.17	0.16	0.39[a]	0.21	1+

Positive and negative correlations are marked in pink and blue, respectively. The colour intensity corresponds with the strength of the correlation. *P*-values: a < 0.05; b < 0.01; c < 0.001; d < 0.0001. Tissues with $P < 0.01$ were regarded as significant. [†]Number of organs with significant ($P < 0.01$) positive (+) or negative (-) correlations. '-' and '*' indicate no gene expression and correlation between same gene, respectively.

Table S13
Pearson's correlation coefficients (R) between mRNA expression levels of *THADA* and PCOS candidate genes in fetal organs and the number of organs with significant ($P < 0.01$) correlations.

Genes	Ovary (N=18)	Testis (N=27)	Heart (N=38)	Kidney (N=29)	Liver (N=37)	Cerebellum (N=33)	Brain (N=32)	Number of organs[†]
age (days)	0.21	-0.12	-0.11	0.01	-0.36[a]	-0.79[d]	-0.78[d]	2-
FSHB	-	-	-	-0.22	-	-0.20	-	
FSHR	0.32	0.14	0.69[d]	-0.05	0.42[b]	0.43[a]	0.47[b]	3+
LHCGR	0.09	0.09	0.33	-0.16	0.23	0.47[b]	0.68[d]	2+
AR	0.27	-0.06	0.42[a]	-0.16	0.03	0.87[d]	0.86[d]	2+
AMH	-0.51[a]	-0.13	-0.59[c]	-0.46[a]	-0.27	-0.38[a]	-0.07	1-
INSR	0.11	0.16	-0.17	0.09	-0.52[c]	-0.16	-0.54[b]	2-
FDFT1	0.64[b]	0.14	0.63[d]	0.07	0.34[a]	0.64[d]	0.10	3+
ERBB3	0.31	0.11	0.68[d]	0.33	0.02	0.51[b]	0.60[c]	3+
ERBB4	0.13	0.41[a]	0.56[c]	0.33	0.40[a]	-0.06	-0.22	1+
PLGRKT	-0.29	-0.40[a]	-0.26	0.23	0.27	0.37[a]	-0.07	
HMGA2	-0.23	0.21	0.53[b]	0.02	0.52[b]	0.87[d]	0.89[d]	4+
TOX3	0.76[c]	0.35	0.40[a]	0.38	0.26	0.50[b]	0.10	2+
GATA4	0.17	0.14	0.11	-0.11	0.45[b]	-0.04	-0.13	1-
YAP1	0.13	0.36	0.74[d]	0.68[c]	0.53[c]	0.93[d]	0.94[d]	5+
ZBTB16	0.74[c]	-0.02	0.21	-0.10	0.39[a]	0.82[d]	0.79[d]	3+
IRF1	0.44	-0.09	0.15	-0.20	-0.29	-0.25	0.10	
NEIL2	0.56[a]	-0.04	0.33	-0.31	0.16	-0.49[b]	-0.48[b]	2-
RAD50	0.83[d]	0.60[b]	0.90[d]	0.56[b]	0.64[d]	0.82[d]	0.65[d]	7+
KRR1	0.48[a]	0.30	0.56[c]	0.52[b]	0.06	0.75[d]	0.58[c]	4+
RAB5B	0.49[a]	0.41[a]	0.70[d]	0.24	0.39[a]	0.25	0.04	1+
ARL14EP	0.29	0.08	0.11	0.39[a]	0.19	0.19	-0.03	
DENND1A	0.30	0.57[b]	0.55[c]	-0.10	0.58[c]	-0.56[c]	0.08	3+, 1-
THADA	*	*	*	*	*	*	*	
MAPRE1	0.85[d]	0.36	0.68[d]	0.42[a]	0.48[b]	0.77[d]	0.73[d]	5+
AOPEP	-0.48[a]	-0.19	-0.72[d]	0.09	-0.50[b]	0.54[b]	0.81[d]	2+, 2-
SUOX	-0.08	-0.39[a]	-0.50[b]	-0.03	-0.09	-0.20	0.08	1-
SUMO1P1	-0.05	-0.08	0.40[a]	-0.13	-0.07	-	-0.23	
FBN3	-0.48[a]	-0.02	0.36[a]	-0.24	0.47[b]	-0.08	-0.09	1+

Positive and negative correlations are marked in pink and blue, respectively. The colour intensity corresponds with the strength of the correlation. *P*-values: a < 0.05; b < 0.01; c < 0.001; d < 0.0001. Tissues with $P < 0.01$ were regarded as significant. [†]Number of organs with significant ($P < 0.01$) positive (+) or negative (-) correlations. '-' and '*' indicate no gene expression and correlation between same gene, respectively.

Table S14

Pearson's correlation coefficients (R) between mRNA expression levels of *MAPRE1* and PCOS candidate genes in fetal organs and the number of organs with significant ($P < 0.01$) correlations.

Genes	Ovary (N=18)	Testis (N=27)	Heart (N=38)	Kidney (N=29)	Liver (N=37)	Cerebellum (N=33)	Brain (N=32)	Number of organs[†]
age (days)	0.06	-0.19	-0.41[a]	-0.61[c]	0.16	-0.78[d]	-0.69[d]	3-
FSHB	-	-	-	-0.35	-	-0.26	-	
FSHR	0.08	0.70[d]	0.58[c]	0.33	0.26	0.22	0.26	2+
LHCGR	0.34	0.62[c]	0.23	0.22	0.05	0.19	0.35	1+
AR	0.13	-0.35	0.38[a]	0.09	0.28	0.53[b]	0.59[c]	2+
AMH	-0.55[a]	0.19	-0.31	0.05	-0.65[d]	-0.22	0.03	1-
INSR	0.06	0.42[a]	-0.32	-0.30	0.06	0.05	-0.34	
FDFT1	0.74[c]	0.67[c]	0.37[a]	0.66[c]	0.27	0.82[d]	0.27	4+
ERBB3	0.39	0.55[b]	0.52[b]	-0.38	-0.21	0.16	0.19	2+
ERBB4	0.26	0.28	0.47[b]	-0.32	0.21	0.15	-0.24	1+
PLGRKT	-0.12	0.09	-0.13	0.06	0.09	0.49[b]	0.03	1+
HMGA2	-0.10	-0.03	0.54[c]	0.61[c]	0.26	0.50[b]	0.58[c]	4+
TOX3	0.56[a]	0.06	0.06	-0.34	0.20	0.77[d]	0.23	1+
GATA4	-0.02	0.56[b]	0.22	0.35	0.11	0.19	-0.03	
YAP1	0.22	-0.06	0.60[c]	0.47[a]	0.37[a]	0.63[d]	0.62[c]	2+, 1-
ZBTB16	0.76[c]	-0.32	-0.17	-0.47[a]	0.59[c]	0.48[b]	0.38[a]	3+
IRF1	0.39	0.18	0.02	-0.64[c]	0.21	-0.50[b]	-0.13	2-
NEIL2	0.50[a]	0.48[a]	0.39[a]	0.32	0.36[a]	-0.48[b]	-0.50[b]	2-
RAD50	0.78[c]	0.51[b]	0.69[d]	0.48[a]	0.75[d]	0.65[d]	0.52[b]	6+
KRR1	0.53[a]	0.06	0.39[a]	0.27	0.58[c]	0.70[d]	0.50[b]	3+
RAB5B	0.54[a]	0.64[c]	0.39[a]	-0.34	0.78[d]	0.27	0.23	2+
ARL14EP	0.32	-0.07	0.10	0.25	0.21	0.43[a]	0.13	
DENND1A	0.09	0.16	0.32	-0.33	0.55[c]	-0.55[c]	-0.02	1+, 1-
THADA	0.85[d]	0.36	0.68[d]	0.42[a]	0.48[b]	0.78[d]	0.73[d]	5+
MAPRE1	*	*	*	*	*	*	*	
AOPEP	-0.38	-0.61[c]	-0.60[c]	-0.49[b]	-0.66[d]	0.08	0.43[a]	4-
SUOX	0.16	-0.28	-0.35[a]	-0.53[b]	-0.63[d]	-0.25	-0.12	2-
SUMO1P1	-0.18	0.05	0.38[a]	0.04	0.02	-	-0.23	
FBN3	-0.52[a]	-0.17	0.40[a]	0.24	0.23	-0.50[b]	-0.37[a]	1-

Positive and negative correlations are marked in pink and blue, respectively. The colour intensity corresponds with the strength of the correlation. *P*-values: a < 0.05; b < 0.01; c < 0.001; d < 0.0001. Tissues with $P < 0.01$ were regarded as significant. [†]Number of organs with significant ($P < 0.01$) positive (+) or negative (-) correlations. '-' and '*' indicate no gene expression and correlation between same gene, respectively.

Table S15

Pearson's correlation coefficients (R) between mRNA expression levels of *AOPEP* and PCOS candidate genes in fetal organs and the number of organs with significant ($P < 0.01$) correlations.

Genes	Ovary (N=18)	Testis (N=27)	Heart (N=38)	Kidney (N=29)	Liver (N=37)	Cerebellum (N=33)	Brain (N=32)	Number of organs[†]
age (days)	0.02	0.11	0.28	0.57[b]	-0.20	-0.41[a]	-0.65[d]	1+, 1-
FSHB	-	-	-	0.40[a]	-	-0.15	-	
FSHR	-0.01	-0.24	-0.66[d]	-0.55[b]	-0.18	0.35[a]	0.57[c]	1+, 2-
LHCGR	-0.06	-0.16	-0.33	-0.46[a]	0.01	0.51[b]	0.91[d]	2+
AR	-0.16	0.09	-0.39[a]	-0.36	-0.23	0.57[c]	0.83[d]	2+
AMH	0.67[b]	0.32	0.39[a]	-0.24	0.51[b]	-0.19	-0.04	2+
INSR	0.37	0.13	0.60[c]	0.25	0.31	-0.39[a]	-0.67[d]	1+, 1-
FDFT1	-0.32	-0.33	-0.44[b]	-0.38[a]	-0.12	0.12	-0.26	1+
ERBB3	-0.43	0.12	-0.65[d]	0.60[c]	-0.15	0.61[c]	0.84[d]	3+, 1-
ERBB4	-0.32	-0.37	-0.34[a]	0.39[a]	-0.16	-0.43[a]	-0.08	
PLGRKT	0.16	0.33	0.45[b]	0.36	0.01	0.12	-0.30	1+
HMGA2	-0.06	-0.20	-0.59[c]	-0.58[b]	-0.25	0.67[d]	0.85[d]	2+, 2-
TOX3	-0.09	-0.28	-0.32	0.60[b]	-0.16	-0.08	0.02	1+
GATA4	0.15	0.10	-0.08	-0.56[b]	-0.18	-0.27	-0.01	1-
YAP1	-0.26	-0.34	-0.47[b]	-0.14	-0.32	0.63[d]	0.91[d]	2+, 1-
ZBTB16	-0.33	-0.07	0.11	0.47[a]	-0.48[b]	0.61[c]	0.91[d]	2+, 1-
IRF1	-0.02	-0.19	-0.10	0.62[c]	-0.19	0.19	0.14	1+
NEIL2	-0.05	-0.08	-0.28	-0.41[a]	0.07	-0.26	-0.20	
RAD50	-0.58[a]	-0.59[b]	-0.63[d]	-0.41[a]	-0.80[d]	0.38[a]	0.45[b]	1+, 3-
KRR1	-0.08	-0.08	-0.20	-0.05	-0.12	0.25	0.44[a]	
RAB5B	-0.66[b]	-0.71[d]	-0.50[b]	0.48[a]	-0.82[d]	-0.11	-0.01	3-
ARL14EP	0.04	0.02	0.18	-0.15	-0.17	-0.33	-0.24	
DENND1A	0.04	0.03	-0.31	0.45[a]	-0.46[b]	-0.26	-0.03	1-
THADA	-0.48[a]	-0.19	-0.72[d]	0.09	-0.50[b]	0.54[b]	0.81[d]	2+, 2-
MAPRE1	-0.38	-0.61[c]	-0.60[c]	-0.49[b]	-0.66[d]	0.08	0.43[a]	4-
AOPEP	*	*	*	*	*	*	*	
SUOX	-0.02	0.22	0.21	0.56[b]	0.35[a]	0.19	0.41[a]	1+
SUMO1P1	0.18	-0.13	-0.35[a]	-0.03	-0.06	-	-0.17	
FBN3	0.52[a]	0.23	-0.54[b]	-0.38[a]	-0.20	0.55[c]	0.11	1+, 1-

Positive and negative correlations are marked in pink and blue, respectively. The colour intensity corresponds with the strength of the correlation. *P*-values: a < 0.05; b < 0.01; c < 0.001; d < 0.0001. Tissues with $P < 0.01$ were regarded as significant. [†]Number of organs with significant ($P < 0.01$) positive (+) or negative (-) correlations. '-' and '*' indicate no gene expression and correlation between same gene, respectively.

Table S16

Pearson's correlation coefficients (R) between mRNA expression levels of *SUOX* and PCOS candidate genes in fetal organs and the number of organs with significant ($P < 0.01$) correlations.

Genes	Ovary (N=18)	Testis (N=27)	Heart (N=38)	Kidney (N=29)	Liver (N=37)	Cerebellum (N=33)	Brain (N=32)	Number of organs[†]
age (days)	-0.26	-0.41[a]	-0.26766	0.12	-0.33[a]	0.16	-0.13	
FSHB	-	-	-	0.11	-	0.25	-	
FSHR	-0.08	-0.12	-0.32	-0.11	-0.20	-0.14	0.30	
LHCGR	0.04	-0.12	-0.17	-0.53[b]	0.02	0.22	0.34	1-
AR	-0.09	-0.31	-0.32	-0.26	-0.21	-0.08	0.43[a]	
AMH	0.00	0.17	0.63[d]	-0.04	0.44[b]	0.18	0.24	2+
INSR	0.09	-0.27	-0.20	0.21	-0.20	-0.39[a]	-0.47[b]	1-
FDFT1	0.09	-0.16	-0.18	-0.26	0.02	0.04	-0.35[a]	
ERBB3	0.23	0.00	-0.21	0.39[a]	0.50[b]	0.07	0.26	1+
ERBB4	0.02	-0.28	-0.58[c]	-0.05	-0.16	-0.45[b]	0.44[a]	2-
PLGRKT	0.37	0.23	0.03	-0.13	-0.04	-0.04	-0.35	
HMGA2	0.12	0.20	-0.04	-0.23	-0.06	-0.01	0.26	
TOX3	-0.20	0.03	0.12	0.23	-0.09	-0.25	0.45[b]	1+
GATA4	0.04	0.07	-0.08	-0.20	0.12	-0.21	0.01	
YAP1	-0.04	-0.37	-0.60[c]	-0.51[b]	-0.15	-0.08	0.27	2-
ZBTB16	-0.05	-0.41[a]	-0.47[b]	0.66[c]	-0.26	-0.03	0.32	1+, 1-
IRF1	0.21	-0.34	-0.33	0.49[b]	-0.30	0.27	0.11	1+
NEIL2	-0.08	-0.10	-0.10	-0.04	-0.49[b]	0.22	0.35	1-
RAD50	-0.12	-0.40[a]	-0.58[c]	-0.55[b]	-0.29	-0.37[a]	-0.13	2-
KRR1	-0.22	-0.46[a]	-0.50[b]	-0.49[b]	-0.50[b]	-0.48[b]	0.09	4-
RAB5B	0.22	-0.40[a]	-0.47[b]	0.38	-0.39[a]	-0.24	0.05	1-
ARL14EP	-0.26	-0.25	-0.35[a]	-0.53[b]	-0.11	-0.46[b]	-0.22	2-
DENND1A	0.07	-0.10	-0.22	0.59[b]	-0.28	0.14	0.08	1+
THADA	-0.08	-0.39[a]	-0.50[b]	-0.03	-0.09	-0.20	0.08	1-
MAPRE1	0.16	-0.28	-0.35[a]	-0.53[b]	-0.63[d]	-0.25	-0.12	2-
AOPEP	-0.02	0.22	0.21	0.56[b]	0.35[a]	0.19	0.41[a]	1-
SUOX	*	*	*	*	*	*	*	
SUMO1P1	0.16	-0.11	-0.02	-0.19	-0.05	-	-0.18	
FBN3	0.08	0.48[a]	0.14	0.06	-0.11	0.30	0.10	

Positive and negative correlations are marked in pink and blue, respectively. The colour intensity corresponds with the strength of the correlation. *P*-values: a < 0.05; b < 0.01; c < 0.001; d < 0.0001. Tissues with $P < 0.01$ were regarded as significant. [†]Number of organs with significant ($P < 0.01$) positive (+) or negative (-) correlations. '-' and '*' indicate no gene expression and correlation between same gene, respectively.

Table S17

Pearson's correlation coefficients (R) between mRNA expression levels of *SUMO1P1* and PCOS candidate genes in fetal organs and the number of organs with significant ($P < 0.01$) correlations.

Genes	Ovary (N=18)	Testis (N=27)	Heart (N=38)	Kidney (N=29)	Liver (N=37)	Cerebellum (N=33)	Brain (N=32)	Number of organs[†]
age (days)	0.09	0.10	-0.18	0.06	0.00	-	0.22	
FSHB	-	-	-	0.12	-	-	-	
FSHR	0.38	0.27	0.23	0.40[a]	-0.13	-	-0.15	
LHCGR	-0.33	0.01	0.03	0.00	0.30	-	-0.18	
AR	-0.08	0.10	0.17	0.59[b]	-0.20	-	-0.15	1+
AMH	0.32	-0.05	-0.03	-0.16	-0.27	-	-0.01	
INSR	0.62[b]	0.13	-0.15	-0.08	-0.15	-	0.00	1+
FDFT1	-0.26	-0.04	0.30	-0.08	0.15	-	-0.22	
ERBB3	0.05	0.05	0.39[a]	0.03	-0.25	-	-0.10	
ERBB4	-0.40	0.03	0.16	0.04	-0.11	-	-0.08	
PLGRKT	-0.23	-0.13	0.01	0.09	0.38[a]	-	0.22	
HMGA2	-0.45	-0.08	0.35[a]	-0.01	-0.13	-	-0.10	
TOX3	0.27	-0.08	0.29	-0.13	0.03	-	-0.21	
GATA4	0.66[b]	0.09	-0.11	0.15	-0.10	-	0.23	1+
YAP1	-0.42	0.05	0.40[a]	-0.06	-0.14	-	-0.16	
ZBTB16	-0.06	0.06	-0.26	-0.13	-0.22	-	-0.20	
IRF1	0.11	0.06	-0.11	0.26	0.14	-	0.27	
NEIL2	0.21	0.02	0.11	0.10	0.03	-	0.25	
RAD50	-0.18	0.15	0.33	0.05	-0.08	-	-0.24	
KRR1	0.23	0.29	0.42[a]	0.03	-0.22	-	-0.10	
RAB5B	-0.52[a]	0.15	0.13	0.17	-0.12	-	-0.23	
ARL14EP	-0.35	0.06	0.28	0.01	0.22	-	-0.09	
DENND1A	0.54[a]	0.08	0.05	-0.06	-0.10	-	-0.29	
THADA	-0.05	-0.08	0.40[a]	-0.13	-0.07	-	-0.23	
MAPRE1	-0.18	0.05	0.38[a]	0.04	0.02	-	-0.23	
AOPEP	0.18	-0.13	-0.35[a]	-0.03	-0.06	-	-0.17	
SUOX	0.16	-0.11	-0.02	-0.19	-0.05	-	-0.18	
SUMO1P1	*	*	*	*	*	*	*	
FBN3	0.53[a]	-0.14	0.37[a]	-0.23	-0.18	-	-0.12	

Positive and negative correlations are marked in pink and blue, respectively. The colour intensity corresponds with the strength of the correlation. *P*-values: a < 0.05; b < 0.01; c < 0.001; d < 0.0001. Tissues with $P < 0.01$ were regarded as significant. [†]Number of organs with significant ($P < 0.01$) positive (+) or negative (-) correlations. '-' and '*' indicate no gene expression and correlation between same gene, respectively.

Table S18

Pearson's correlation coefficients (R) between mRNA expression levels of *ERBB3* and PCOS candidate genes in fetal organs and the number of organs with significant ($P < 0.01$) correlations.

Genes	Ovary (N=18)	Testis (N=27)	Heart (N=38)	Kidney (N=29)	Liver (N=37)	Cerebellum (N=33)	Brain (N=32)	Number of organs[†]
age (days)	-0.31	0.05	-0.40[a]	0.52[b]	-0.04	-0.34	-0.38[a]	1+
FSHB	-	-	-	0.00	-	-0.08	-	
FSHR	0.36	0.68[c]	0.88[d]	-0.53[b]	-0.10	0.56[c]	0.54[b]	4+, 1-
LHCGR	0.08	0.53[b]	0.60[c]	-0.45[a]	0.01	0.52[b]	0.87[d]	4+
AR	0.08	-0.18	0.49[b]	-0.36	0.17	0.59[c]	0.58[c]	3+
AMH	-0.22	0.59[b]	-0.34[a]	-0.55[b]	0.09	-0.38[a]	-0.22	1+, 1-
INSR	-0.06	0.67[c]	-0.40[a]	0.20	-0.12	-0.49[b]	-0.44[a]	1+, 1-
FDFT1	0.05	0.46[a]	0.74[d]	-0.40[a]	0.12	0.11	-0.28	1+
ERBB3		*	*	*	*	*	*	
ERBB4	0.22	-0.15	0.05	0.59[b]	-0.06	-0.39[a]	-0.08	1+
PLGRKT	0.28	0.41[a]	-0.54[c]	0.35	-0.12	-0.09	-0.37[a]	1-
HMGA2	0.05	-0.37	0.82[d]	-0.68[d]	0.10	0.68[d]	0.60[c]	3+, 1-
TOX3	0.16	-0.36	0.55[c]	0.51[b]	0.07	-0.14	-0.14	2+
GATA4	0.15	0.76[d]	0.07	-0.48[a]	0.18	-0.09	-0.10	1+
YAP1	0.22	-0.45[a]	0.37[a]	0.03	0.09	0.65[d]	0.73[d]	2+
ZBTB16	0.06	-0.36	-0.15	0.37	0.14	0.83[d]	0.88[d]	2+
IRF1	0.01	0.14	0.20	0.48[a]	0.06	0.26	0.13	
NEIL2	-0.13	0.62[c]	0.56[c]	-0.60[c]	-0.39[a]	-0.06	-0.05	2+,1-
RAD50	0.32	-0.01	0.50[b]	-0.21	0.12	0.40[a]	0.42[a]	1+
KRR1	0.19	-0.05	0.18	-0.02	-0.33[a]	0.26	0.38[a]	
RAB5B	0.43	0.08	0.33	0.63[c]	0.25	0.08	0.05	1+
ARL14EP	0.27	-0.02	-0.12	-0.07	-0.01	-0.25	-0.20	
DENND1A	0.12	-0.07	0.48[b]	0.34	-0.10	-0.19	0.01	1+
THADA	0.31	0.11	0.68[d]	0.33	0.02	0.51[b]	0.60[c]	3+
MAPRE1	0.39	0.55[b]	0.52[b]	-0.38	-0.21	0.16	0.19	2+
AOPEP	-0.43	0.12	-0.65[d]	0.60[c]	-0.15	0.61[c]	0.86[d]	3+, 1-
SUOX	0.23	0.00	-0.21	0.39[a]	0.50[b]	0.07	0.26	1+
SUMO1P1	0.05	0.05	0.39[a]	0.03	-0.25	-	-0.10	
FBN3	-0.12	-0.17	0.70[d]	-0.57[b]	0.01	0.43[a]	0.35	1+, 1-

Positive and negative correlations are marked in pink and blue, respectively. The colour intensity corresponds with the strength of the correlation. *P*-values: a < 0.05; b < 0.01; c < 0.001; d < 0.0001. Tissues with $P < 0.01$ were regarded as significant. [†]Number of organs with significant ($P < 0.01$) positive (+) or negative (-) correlations. '-' and '*' indicate no gene expression and correlation between same gene, respectively.

Table S19

Pearson's correlation coefficients (R) between mRNA expression levels of *ERBB4* and PCOS candidate genes in fetal organs and the number of organs with significant ($P < 0.01$) correlations.

Genes	Ovary (N=18)	Testis (N=27)	Heart (N=38)	Kidney (N=29)	Liver (N=37)	Cerebellum (N=33)	Brain (N=32)	Number of organs[†]
age (days)	-0.61[b]	-0.47[a]	0.12	0.80[d]	-0.27	0.23	0.29	1+, 1-
FSHB	-	-	-	0.13	-	0.18	-	
FSHR	-0.45	-0.01	0.17	-0.40[a]	0.97[d]	-0.29	0.02	1+
LHCGR	0.87[d]	-0.27	-0.08	-0.24	0.27	-0.49[b]	-0.07	1+,1-
AR	-0.35	-0.27	0.04	-0.06	0.05	-0.27	0.09	
AMH	-0.37	-0.48[a]	-0.43[a]	-0.77[d]	0.04	-0.31	-0.14	1-
INSR	-0.21	-0.40[a]	0.25	0.08	-0.33[a]	0.78[d]	0.03	1+
FDFT1	0.37	-0.23	0.09	-0.51[b]	-0.11	-0.08	-0.35[a]	1+
ERBB3	0.22	-0.15	0.05	0.59[b]	-0.06	-0.39[a]	-0.08	1+
ERBB4	*	*	*	*	*	*	*	
PLGRKT	0.55[a]	-0.23	-0.05	0.69[d]	-0.21	-0.21	-0.37[a]	1+
HMGA2	0.77[c]	0.61[c]	0.11	-0.74[d]	0.78[d]	-0.32	-0.22	2+, 2-
TOX3	-0.35	0.56[b]	-0.23	0.63[c]	0.32	0.33	0.65[d]	3+
GATA4	-0.77[c]	-0.20	0.18	-0.43[a]	0.63[d]	0.21	0.02	1+, 1-
YAP1	0.93[d]	0.67[c]	0.82[d]	0.49[b]	0.83[d]	-0.25	-0.08	4+
ZBTB16	-0.06	-0.07	0.36[a]	-0.01	0.14	-0.26	0.01	
IRF1	-0.60[b]	-0.44[a]	0.11	0.32	-0.23	-0.25	0.08	1-
NEIL2	-0.55[a]	-0.39[a]	0.00	-0.77[d]	0.01	0.03	0.26	1-
RAD50	0.11	0.53[b]	0.66[d]	0.30	0.31	0.25	0.02	2+
KRR1	0.15	0.18	0.63[d]	0.54[b]	0.04	0.38[a]	0.39[a]	2+
RAB5B	0.51[a]	0.27	0.53[b]	0.40[a]	0.18	0.54[b]	0.50[b]	3+
ARL14EP	0.55[a]	0.03	0.37[a]	0.52[b]	0.33[a]	0.74[d]	0.04	2+
DENND1A	-0.58[a]	0.09	0.20	-0.20	0.13	0.01	-0.13	
THADA	0.13	0.41[a]	0.56[c]	0.33	0.40[a]	-0.06	-0.22	1+
MAPRE1	0.26	0.28	0.47[b]	-0.32	0.21	0.15	-0.24	1+
AOPEP	-0.32	-0.37	-0.34[a]	0.39[a]	-0.16	-0.43[a]	-0.08	
SUOX	0.02	-0.28	-0.58[c]	-0.05	-0.16	-0.45[b]	0.44[a]	2-
SUMO1P1	-0.40	0.03	0.16	0.04	-0.11	-	-0.08	
FBN3	-0.26	0.33	-0.08	-0.79[d]	0.78[d]	-0.42[a]	0.10	1+, 1-

Positive and negative correlations are marked in pink and blue, respectively. The colour intensity corresponds with the strength of the correlation. *P*-values: a < 0.05; b < 0.01; c < 0.001; d < 0.0001. Tissues with $P < 0.01$ were regarded as significant. [†]Number of organs with significant ($P < 0.01$) positive (+) or negative (-) correlations. '-' and '*' indicate no gene expression and correlation between same gene, respectively.

Table S20

Pearson's correlation coefficients (R) between mRNA expression levels of *PLGRKT* and PCOS candidate genes in fetal organs and the number of organs with significant ($P < 0.01$) correlations.

Genes	Ovary (N=18)	Testis (N=27)	Heart (N=38)	Kidney (N=29)	Liver (N=37)	Cerebellum (N=33)	Brain (N=32)	Number of organs[†]
age (days)	-0.72[c]	-0.32	0.53[b]	0.52[b]	0.14	-0.48[b]	-0.15	2+, 2-
FSHB	-	-	-	0.16	-	-0.19	-	
FSHR	-0.15	0.14	-0.59[c]	-0.25	-0.20	0.00	-0.31	1-
LHCGR	0.57[a]	0.32	-0.31	-0.21	0.25	0.03	-0.34	
AR	-0.37	-0.48[a]	-0.11	0.04	0.19	0.16	-0.19	
AMH	0.37	0.61[c]	-0.02	-0.67[c]	-0.35[a]	0.45[b]	0.48[b]	3+, 1-
INSR	-0.19	0.29	0.10	0.03	-0.11	-0.11	-0.18	
FDFT1	-0.11	0.16	-0.27	-0.28	0.41[a]	0.70[d]	0.46[b]	2+
ERBB3	0.28	0.41[a]	-0.54[c]	0.35	-0.12	-0.09	-0.37[a]	1-
ERBB4	0.55[a]	-0.23	-0.05	0.69[d]	-0.21	-0.21	-0.37[a]	1+
PLGRKT	*	*	*	*	*	*	*	
HMGA2	0.63[b]	-0.09	-0.60[c]	-0.42[a]	-0.22	0.19	-0.10	1+, 1-
TOX3	-0.47[a]	-0.29	-0.26	0.44[a]	-0.17	0.44[b]	-0.30	1+
GATA4	-0.41	0.45[a]	-0.42[a]	-0.28	-0.24	-0.14	0.07	
YAP1	0.51[a]	-0.59[b]	-0.03	0.54[b]	-0.24	0.25	-0.21	1+, 1-
ZBTB16	-0.54[a]	-0.55[b]	0.02	-0.07	-0.01	0.02	-0.38[a]	1-
IRF1	-0.59[a]	-0.29	-0.11	0.17	0.22	-0.49[b]	-0.04	1-
NEIL2	-0.72[c]	0.20	-0.43[a]	-0.51[b]	0.47[b]	-0.40[a]	-0.25	1+, 2-
RAD50	-0.39	-0.50[b]	-0.17	0.31	-0.08	-0.02	-0.43[a]	1-
KRR1	-0.20	-0.25	0.24	0.53[b]	-0.06	0.14	-0.30	1+
RAB5B	0.05	-0.59[b]	-0.11	0.23	-0.07	-0.47[b]	-0.66[d]	3-
ARL14EP	0.30	-0.05	0.55[c]	0.54[b]	0.06	0.09	-0.15	2+
DENND1A	-0.36	-0.31	-0.47[b]	-0.23	0.05	-0.56[c]	-0.26	2-
THADA	-0.29	-0.40[a]	-0.26	0.23	0.27	0.37[a]	-0.07	
MAPRE1	-0.12	0.09	-0.13	0.06	0.09	0.49[b]	0.03	1+
AOPEP	0.16	0.33	0.45[b]	0.36	0.01	0.12	-0.30	1+
SUOX	0.37	0.23	0.03	-0.13	-0.04	-0.04	-0.35	
SUMO1P1	-0.23	-0.13	0.01	0.09	0.38[a]	-	0.22	
FBN3	0.20	0.11	-0.63[d]	-0.59[b]	-0.25	-0.21	-0.43[a]	2-

Positive and negative correlations are marked in pink and blue, respectively. The colour intensity corresponds with the strength of the correlation. *P*-values: a < 0.05; b < 0.01; c < 0.001; d < 0.0001. Tissues with $P < 0.01$ were regarded as significant. [†]Number of organs with significant ($P < 0.01$) positive (+) or negative (-) correlations. '-' and '*' indicate no gene expression and correlation between same gene, respectively.

Table S21

Pearson's correlation coefficients (R) between mRNA expression levels of *FBN3* and PCOS candidate genes in fetal organs and the number of organs with significant ($P < 0.01$) correlations.

Genes	Ovary (N=18)	Testis (N=27)	Heart (N=38)	Kidney (N=29)	Liver (N=37)	Cerebellum (N=33)	Brain (N=32)	Number of organs[†]
age (days)	-0.44	-0.73[d]	-0.77[d]	-0.73[d]	-0.46[b]	0.17	0.43[a]	4-
FSHB	-	-	-	-0.28	-	0.20	-	
FSHR	0.24	-0.25	0.62[d]	0.32	0.85[d]	0.01	0.10	2+
LHCGR	-0.22	-0.44[a]	0.21	0.22	0.28	0.20	0.12	
AR	-0.34	-0.62[c]	0.03	-0.02	-0.02	0.07	-0.07	1-
AMH	0.74[c]	-0.21	0.13	0.80[d]	0.07	0.09	-0.38[a]	2+
INSR	0.50[a]	-0.45[a]	-0.40[a]	-0.21	-0.52[c]	-0.25	0.28	
FDFT1	-0.60[b]	-0.42[a]	0.50[b]	0.28	-0.06	-0.45[b]	-0.16	1+, 2-
ERBB3	-0.12	-0.17	0.70[d]	-0.57[b]	0.01	0.43[a]	0.35	1+, 1-
ERBB4	-0.26	0.33	-0.08	-0.79[d]	0.78[d]	-0.42[a]	0.10	1+, 1-
PLGRKT	0.20	0.11	-0.63[d]	-0.59[b]	-0.25	-0.21	-0.43[a]	2-
HMGA2	-0.02	0.75[d]	0.87[d]	0.73[d]	0.95[d]	0.26	-0.01	4+
TOX3	-0.05	0.62[c]	0.51[b]	-0.58[b]	0.56[c]	-0.51[b]	0.02	3+, 2-
GATA4	0.30	0.09	0.39[a]	0.25	0.86[d]	-0.20	-0.09	1+
YAP1	-0.20	0.13	-0.02	-0.29	0.92[d]	0.08	0.02	1+
ZBTB16	-0.50[a]	-0.36	-0.33	-0.07	0.18	0.23	0.24	
IRF1	-0.35	-0.75[d]	0.09	-0.51[b]	-0.38[a]	0.42[a]	-0.02	2-
NEIL2	-0.33	-0.43[a]	0.52[b]	0.52[b]	-0.12	0.18	0.27	2+
RAD50	-0.62[b]	-0.10	0.14	-0.17	0.33[a]	-0.16	0.20	1-
KRR1	-0.10	-0.12	-0.17	-0.39[a]	-0.02	-0.22	-0.01	
RAB5B	-0.71[b]	-0.30	-0.11	-0.43[a]	0.16	-0.36[a]	0.23	1-
ARL14EP	-0.10	-0.18	-0.44[b]	-0.43[a]	0.33[a]	-0.45[b]	0.16	2-
DENND1A	0.24	0.18	0.47[b]	0.17	0.16	0.39[a]	0.21	1+
THADA	-0.48[a]	-0.02	0.36[a]	-0.24	0.47[b]	-0.08	-0.09	1+
MAPRE1	-0.52[a]	-0.17	0.40[a]	0.24	0.23	-0.50[b]	-0.37[a]	1-
AOPEP	0.52[a]	0.23	-0.54[b]	-0.38[a]	-0.20	0.55[c]	0.11	1+, 1-
SUOX	0.08	0.48[a]	0.14	0.06	-0.11	0.30	0.10	
SUMO1P1	0.53[a]	-0.14	0.37[a]	-0.23	-0.18		-0.12	
FBN3	*	*	*	*	*	*	*	

Positive and negative correlations are marked in pink and blue, respectively. The colour intensity corresponds with the strength of the correlation. *P*-values: a < 0.05; b < 0.01; c < 0.001; d < 0.0001. Tissues with $P < 0.01$ were regarded as significant. [†]Number of organs with significant ($P < 0.01$) positive (+) or negative (-) correlations. '-' and '*' indicate no gene expression and correlation between same gene, respectively.

Table S22

Pearson's correlation coefficients (R) between mRNA expression levels of *INSR* and PCOS candidate genes in fetal organs and the number of organs with significant ($P < 0.01$) correlations.

Genes	Ovary (N=18)	Testis (N=27)	Heart (N=38)	Kidney (N=29)	Liver (N=37)	Cerebellum (N=33)	Brain (N=32)	Number of organs[†]
age (days)	-0.14	0.36	0.29	0.31	0.52[b]	0.20	0.74[d]	2+
FSHB	-	-	-	0.28	-	0.02	-	
FSHR	0.07	0.65[c]	-0.35[a]	-0.12	-0.34[a]	-0.44[a]	-0.46[b]	1+, 1-
LHCGR	-0.18	0.84[d]	-0.32	0.17	-0.36[a]	-0.45[b]	-0.61[c]	1+, 2-
AR	0.01	-0.01	-0.31	0.15	0.24	-0.42[a]	-0.69[d]	1-
AMH	0.12	0.73[d]	-0.12	-0.16	-0.03	-0.170	-0.37[a]	1+
INSR	1.00	*	*	*	*	*	*	
FDFT1	-0.15	0.75[d]	-0.17	-0.23	-0.30	-0.20	0.09	1+
ERBB3	-0.06	0.67[c]	-0.40[a]	0.20	-0.12	-0.49[b]	-0.44[a]	1+, 1-
ERBB4	-0.21	-0.40[a]	0.25	0.08	-0.33[a]	0.78[d]	0.03	1+
PLGRKT	-0.19	0.29	0.10	0.03	-0.11	-0.11	-0.18	
HMGA2	-0.16	-0.71[d]	-0.33	-0.22	-0.61[d]	-0.42[a]	-0.65[d]	3+
TOX3	050[a]	-0.55[b]	-0.26	0.62[c]	-0.24	0.33	-0.10	1+, 1-
GATA4	0.35	0.73[d]	0.24	0.01	-0.68[d]	0.23	-0.17	1+, 1-
YAP1	-0.10	-0.58[b]	0.10	-0.07	-0.50[b]	-0.39[a]	-0.68[d]	3-
ZBTB16	0.15	-0.02	0.55[c]	0.43[a]	0.16	-0.39[a]	-0.57[c]	1+, 1-
IRF1	0.08	0.42[a]	0.21	0.42[a]	0.46[b]	-0.21	-0.14	1+
NEIL2	0.19	0.71[d]	0.00	0.06	0.28	-0.22	0.22	1+
RAD50	-0.06	-0.10	0.01	0.00	-0.14	0.17	0.09	
KRR1	0.51[a]	-0.05	0.24	-0.02	0.51[b]	0.32	-0.19	1+
RAB5B	-0.39	0.17	0.13	0.36	0.01	0.26	0.40[a]	
ARL14EP	0.18	-0.13	0.27	0.14	-0.08	0.74[d]	0.53[b]	2+
DENND1A	0.49[a]	0.26	0.23	0.13	-0.15	0.07	0.28	
THADA	0.11	0.16	-0.17	0.09	-0.52[c]	-0.16	-0.54[b]	2-
MAPRE1	0.06	0.42[a]	-0.32	-0.30	0.06	0.05	-0.34	
AOPEP	0.37	0.13	0.60[c]	0.25	0.31	-0.39[a]	-0.67[d]	1+, 1-
SUOX	0.09	-0.27	-0.20	0.21	-0.20	-0.39[a]	-0.47[b]	1-
SUMO1P1	0.62[b]	0.13	-0.15	-0.08	-0.15	-	0.00	1+
FBN3	0.50[a]	-0.45[a]	-0.40[a]	-0.21	-0.52[c]	-0.25	0.28	1-

Positive and negative correlations are marked in pink and blue, respectively. The colour intensity corresponds with the strength of the correlation. *P*-values: a < 0.05; b < 0.01; c < 0.001; d < 0.0001. Tissues with $P < 0.01$ were regarded as significant. [†]Number of organs with significant ($P < 0.01$) positive (+) or negative (-) correlations. '-' and '*' indicate no gene expression and correlation between same gene, respectively.

Table S23

Pearson's correlation coefficients (R) between mRNA expression levels of *FDFT1* and PCOS candidate genes in fetal organs and the number of organs with significant ($P < 0.01$) correlations.

Genes	Ovary (N=18)	Testis (N=27)	Heart (N=38)	Kidney (N=29)	Liver (N=37)	Cerebellum (N=33)	Brain (N=32)	Number of organs[†]
age (days)	0.20	0.17	-0.26	-0.57[b]	-0.42[a]	-0.72[d]	-0.14	2-
FSHB	-	-		-0.25	-	-0.17	-	
FSHR	-0.04	0.69[c]	0.56[c]	0.27	-0.13	0.08	-0.10	2+
LHCGR	0.50[a]	0.92[d]	0.14	-0.04	0.52[b]	0.11	-0.25	2+
AR	-0.18	-0.16	0.02	-0.13	-0.26	0.42[a]	-0.11	
AMH	-0.50[a]	0.61[c]	-0.33	0.23	-0.41[a]	0.14	0.27	1+
INSR	-0.15	0.75[d]	-0.17	-0.23	-0.30	-0.20	0.09	1+
FDFT1	*	*	*	*	*	*	*	
ERBB3	0.05	0.47[a]	0.74[d]	-0.40[a]	0.12	0.11	-0.28	1+
ERBB4	0.37	-0.23	0.09	-0.52[b]	-0.11	-0.08	-0.35[a]	1-
PLGRKT	-0.11	0.16	-0.27	-0.28	0.41[a]	0.70[d]	0.46[b]	2+
HMGA2	-0.07	-0.50[b]	0.75[d]	0.57[b]	0.15	0.41[a]	-0.18	2+, 1-
TOX3	0.27	-0.41[a]	0.81[d]	-0.38[a]	0.31	0.72[d]	0.05	2+
GATA4	-0.18	0.65[c]	0.04	0.38[a]	0.24	0.06	-0.02	1+
YAP1	0.17	-0.53[b]	0.36[a]	-0.05	0.15	0.52[b]	-0.09	1+, 1-
ZBTB16	0.67[b]	-0.16	0.12	-0.19	0.05	0.40[a]	-0.18	1+
IRF1	0.32	0.51[b]	0.24	-0.41[a]	-0.27	-0.59[c]	-0.35[a]	1+, 1-
NEIL2	0.47[a]	0.76[d]	0.37[a]	0.49[b]	0.16	-0.45[b]	-0.18	2+, 1-
RAD50	0.42	0.03	0.47[b]	0.05	0.21	0.36[a]	-0.13	1+
KRR1	0.14	-0.31	0.18	-0.17	-0.08	0.36[a]	-0.14	
RAB5B	0.55[a]	0.40[a]	0.29	-0.14	0.20	-0.05	-0.22	
ARL14EP	-0.01	-0.31	-0.05	-0.24	0.13	0.12	0.21	
DENND1A	0.07	0.28	0.64[d]	0.09	0.33[a]	-0.63[d]	0.32	1+, 1-
THADA	0.64[b]	0.14	0.63[d]	0.07	0.34[a]	0.64[d]	0.10	3+
MAPRE1	0.74[c]	0.67[c]	0.37[a]	0.66[c]	0.27	0.82[d]	0.27	4+
AOPEP	-0.32	-0.33	-0.44[b]	-0.38[a]	-0.12	0.12	-0.26	1-
SUOX	0.09	-0.16	-0.18	-0.26	0.02	0.04	-0.35[a]	
SUMO1P1	-0.26	-0.04	0.30	-0.08	0.15	-	-0.22	
FBN3	-0.60[b]	-0.42[a]	0.50[b]	0.28	-0.06	-0.45[b]	-0.16	1+, 2-

Positive and negative correlations are marked in pink and blue, respectively. The colour intensity corresponds with the strength of the correlation. *P*-values: a < 0.05; b < 0.01; c < 0.001; d < 0.0001. Tissues with $P < 0.01$ were regarded as significant. [†]Number of organs with significant ($P < 0.01$) positive (+) or negative (-) correlations. '-' and '*' indicate no gene expression and correlation between same gene, respectively.

Table S24

Pearson's correlation coefficients (R) between mRNA expression levels of *FSHB* and PCOS candidate genes in fetal organs and the number of organs with significant ($P < 0.01$) correlations.

Genes	Ovary (N=18)	Testis (N=27)	Heart (N=38)	Kidney (N=29)	Liver (N=37)	Cerebellum (N=33)	Brain (N=32)	Number of organs[†]
age (days)	-	-	-	0.46[a]	-	0.35[a]	-	
FSHB	*	*	*	*	*	*	*	
FSHR	-	-	-	-0.14	-	-0.06	-	
LHCGR	-	-	-	-0.03	-	-0.11	-	
AR	-	-	-	0.22	-	-0.12	-	
AMH	-	-	-	-0.11	-	0.07	-	
INSR	-	-	-	0.28	-	0.02	-	
FDFT1	-	-	-	-0.25	-	-0.17	-	
ERBB3	-	-	-	0.00	-	-0.08	-	
ERBB4	-	-	-	0.13	-	0.18	-	
PLGRKT	-	-	-	0.16	-	-0.19	-	
HMGA2	-	-	-	-0.34	-	-0.09	-	
TOX3	-	-	-	0.45[a]	-	-0.22	-	
GATA4	-	-	-	-0.14	-	-0.11	-	
YAP1	-	-	-	-0.19	-	-0.12	-	
ZBTB16	-	-	-	0.58[b]	-	-0.12	-	1+
IRF1	-	-	-	0.60[c]	-	0.01	-	1+
NEIL2	-	-	-	0.04	-	0.28	-	
RAD50	-	-	-	-0.19	-	-0.16	-	
KRR1	-	-	-	-0.04	-	-0.18	-	
RAB5B	-	-	-	0.27	-	-0.15	-	
ARL14EP	-	-	-	-0.01	-	-0.03	-	
DENND1A	-	-	-	0.14	-	0.08	-	
THADA	-	-	-	-0.22	-	-0.20	-	
MAPRE1	-	-	-	-0.35	-	-0.27	-	
AOPEP	-	-	-	0.40[a]	-	-0.15	-	
SUOX	-	-	-	0.11	-	0.25	-	
SUMO1P1	-	-	-	0.12	-	-	-	
FBN3	-	-	-	-0.28	-	0.20	-	

Positive and negative correlations are marked in pink and blue, respectively. The colour intensity corresponds with the strength of the correlation. *P*-values: a < 0.05; b < 0.01; c < 0.001; d < 0.0001. Tissues with $P < 0.01$ were regarded as significant. [†]Number of organs with significant ($P < 0.01$) positive (+) or negative (-) correlations. '-' and '*' indicate no gene expression and correlation between same gene, respectively.

Table S25

Pearson's correlation coefficients (R) between mRNA expression levels of *FSHR* and PCOS candidate genes in fetal organs and the number of organs with significant ($P < 0.01$) correlations.

Genes	Ovary (N=18)	Testis (N=27)	Heart (N=38)	Kidney (N=29)	Liver (N=37)	Cerebellum (N=33)	Brain (N=32)	Number of organs[†]
age (days)	0.24	0.16	-0.41[a]	-0.50[b]	-0.28	-0.30	-0.45[b]	2-
FSHB	-	-	-	-0.14	-	-0.06	-	-
FSHR	*	*	*	*	*	*	*	
LHCGR	-0.39	0.74[d]	0.68[d]	0.00	0.26	0.73[d]	0.59[c]	4+
AR	0.16	-0.11	0.61[c]	0.56[b]	0.03	0.64[d]	0.67[d]	4+
AMH	0.35	0.40[a]	-0.35[a]	0.05	0.03	-0.35[a]	-0.08	
INSR	0.07	0.64[c]	-0.35[a]	-0.12	-0.34[a]	-0.44[a]	-0.46[b]	1+, 1-
FDFT1	-0.04	0.69[c]	0.56[c]	0.27	-0.13	0.08	-0.10	2+
ERBB3	0.36	0.68[c]	0.88[d]	-0.53[b]	-0.10	0.56[c]	0.54[b]	4+, 1-
ERBB4	-0.45	-0.01	0.17	-0.40[a]	0.97[d]	-0.29	0.02	1+
PLGRKT	-0.15	0.14	-0.59[c]	-0.25	-0.20	0.00	-0.31	1-
HMGA2	-0.61[b]	-0.37	0.67[d]	0.64[c]	0.82[d]	0.59[c]	0.45[b]	5+, 1-
TOX3	0.47[a]	-0.21	0.37[a]	-0.31	0.34[a]	-0.17	0.08	
GATA4	0.79[d]	0.64[c]	0.20	0.59[b]	0.66[d]	-0.14	0.03	4+
YAP1	-0.45	-0.35	0.41[a]	0.01	0.85[d]	0.62[c]	0.63[c]	3+
ZBTB16	0.15	-0.21	-0.17	-0.12	0.14	0.47[b]	0.77[d]	2+
IRF1	0.39	0.42[a]	0.18	-0.30	-0.21	0.20	0.11	
NEIL2	0.36	0.63[c]	0.58[c]	0.41[a]	0.05	-0.09	-0.12	2+
RAD50	0.10	0.21	0.57[c]	0.31	0.32	0.41[a]	0.38[a]	1+
KRR1	-0.06	0.06	0.17	-0.06	0.04	0.38[a]	0.27	
RAB5B	-0.17	0.48[a]	0.39[a]	-0.11	0.19	0.09	0.05	
ARL14EP	-0.21	-0.13	-0.16	-0.04	0.32	-0.05	-0.26	
DENND1A	0.68[b]	0.14	0.50[b]	-0.09	0.20	-0.34	-0.10	2+
THADA	0.32	0.14	0.69[d]	-0.05	0.42[b]	0.43[a]	0.47[b]	3+
MAPRE1	0.08	0.70[d]	0.58[c]	0.33	0.26	0.22	0.26	2+
AOPEP	-0.01	-0.24	-0.66[d]	-0.55[b]	-0.18	0.35[a]	0.57[c]	1+, 2-
SUOX	-0.08	-0.12	-0.32	-0.11	-0.20	-0.14	0.30	
SUMO1P1	0.38	0.27	0.23	0.40[a]	-0.13	-	-0.15	
FBN3	0.24	-0.25	0.62[d]	0.32	0.85[d]	0.01	0.10	2+

Positive and negative correlations are marked in pink and blue, respectively. The colour intensity corresponds with the strength of the correlation. *P*-values: a < 0.05; b < 0.01; c < 0.001; d < 0.0001. Tissues with $P < 0.01$ were regarded as significant. [†]Number of organs with significant ($P < 0.01$) positive (+) or negative (-) correlations. '-' and '*' indicate no gene expression and correlation between same gene, respectively.

Table S26

Pearson's correlation coefficients (R) between mRNA expression levels of *LHCGR* and PCOS candidate genes in fetal organs and the number of organs with significant ($P < 0.01$) correlations.

Genes	Ovary (N=18)	Testis (N=27)	Heart (N=38)	Kidney (N=29)	Liver (N=37)	Cerebellum (N=33)	Brain (N=32)	Number of organs[†]
age (days)	-0.42	0.20	-0.03	-0.12	-0.46^{b}	-0.37^{a}	-0.61^{c}	2-
FSHB	-	-	-	-0.03	-	-0.110	-	
FSHR	-0.39	0.74^{d}	0.68^{d}	0.00	0.26435	0.732^{d}	0.58^{c}	4+
LHCGR	*	*	*	*	*	*	*	
AR	-0.43	-0.12	0.81^{d}	0.36	-0.18	0.65^{d}	0.70^{d}	3+
AMH	-0.17	0.65^{c}	-0.20	0.36	-0.05	-0.34	-0.02	1+
INSR	-0.18	0.84^{d}	-0.32	0.17	-0.36^{a}	-0.45^{b}	-0.61^{c}	1+, 2-
FDFT1	0.50^{a}	0.92^{d}	0.14	-0.04	0.52^{b}	0.11	-0.25	2+
ERBB3	0.08	0.53^{b}	0.60^{c}	-0.45^{a}	0.01	0.52^{b}	0.87^{d}	4+
ERBB4	0.87^{d}	-0.27	-0.08	-0.24	0.27	-0.49^{b}	-0.07	1+, 1-
PLGRKT	0.57^{a}	0.32	-0.31	-0.21	0.25	0.03	-0.34	
HMGA2	0.52^{a}	-0.58^{b}	0.18	0.13	0.41^{a}	0.68^{d}	0.68^{d}	2+, 1-
TOX3	-0.27	-0.45^{a}	0.06	-0.30	0.54^{c}	-0.22	0.01	1+
GATA4	-0.64^{b}	0.66^{c}	-0.19	0.05	0.42^{a}	-0.19	0.05	1+, 1-
YAP1	0.71^{c}	-0.57^{b}	0.21	0.05	0.41^{a}	0.69^{d}	0.80^{d}	3+, 1-
ZBTB16	0.03	-0.17	-0.25	-0.39^{a}	0.04	0.53^{b}	0.89^{d}	2+
IRF1	-0.53^{a}	0.48^{a}	-0.01	-0.28	-0.24	0.29	0.07	
NEIL2	-0.34	0.73^{d}	0.30	0.26	-0.10	-0.19	-0.20	1+
RAD50	0.05	0.00	0.29	0.20	0.04	0.39^{a}	0.38^{a}	
KRR1	0.23	-0.21	0.19	0.20	-0.19	0.28	0.37^{a}	
RAB5B	0.29	0.33	0.19	-0.46^{a}	0.03	0.01	0.02	
ARL14EP	0.45	-0.20	0.13	0.35	0.42^{a}	-0.15	-0.28	
DENND1A	-0.51^{a}	0.24	-0.06	-0.49^{b}	-0.08	-0.37^{a}	0.00	1-
THADA	0.09	0.09	0.33	-0.16	0.23	0.47^{b}	0.68^{d}	2+
MAPRE1	0.34	0.62^{c}	0.23	0.22	0.05	0.19	0.35	1+
AOPEP	-0.06	-0.16	-0.33	-0.46^{a}	0.01	0.51^{b}	0.91^{d}	2+
SUOX	0.04	-0.12	-0.17	-0.53^{b}	0.02	0.22	0.34	1-
SUMO1P1	-0.33	0.01	0.03	0.00	0.30	-	-0.18	
FBN3	-0.22	-0.44^{a}	0.21	0.22	0.28	0.20	0.12	

Positive and negative correlations are marked in pink and blue, respectively. The colour intensity corresponds with the strength of the correlation. *P*-values: a < 0.05; b < 0.01; c < 0.001; d < 0.0001. Tissues with $P < 0.01$ were regarded as significant. [†]Number of organs with significant ($P < 0.01$) positive (+) or negative (-) correlations. '-' and '*' indicate no gene expression and correlation between same gene, respectively.

Table S27

Pearson's correlation coefficients (R) between mRNA expression levels of *AR* and PCOS candidate genes in fetal organs and the number of organs with significant ($P < 0.01$) correlations.

Genes	Ovary (N=18)	Testis (N=27)	Heart (N=38)	Kidney (N=29)	Liver (N=37)	Cerebellum (N=33)	Brain (N=32)	Number of organs[†]
age (days)	0.51[a]	0.85[d]	0.16	0.09	0.65[d]	-0.58[c]	-0.75[d]	2+, 2-
FSHB	-	-	-	0.22	-	-0.16	-	
FSHR	0.16	-0.11	0.61[c]	0.56[b]	0.03	0.64[d]	0.67[d]	4+
LHCGR	-0.43	-0.12	0.81[d]	0.36	-0.18	0.65[d]	0.70[d]	3+
AR	*	*	*	*	*	*	*	
AMH	-0.26	-0.34	-0.36[a]	-0.06	-0.27	-0.43[a]	0.03	
INSR	0.01	-0.01	-0.31	0.15	0.24	-0.42[a]	-0.69[d]	1-
FDFT1	-0.18	-0.16	0.02	-0.13	-0.26	0.42[a]	-0.11	
ERBB3	0.08	-0.18	0.49[b]	-0.36	0.17	0.59[c]	0.58[c]	3+
ERBB4	-0.35	-0.27	0.04	-0.06	0.05	-0.27	0.09	
PLGRKT	-0.37	-0.48[a]	-0.11	0.04	0.19	0.16	-0.19	
HMGA2	-0.31	-0.49[a]	-0.03	0.19	-0.05	0.92[d]	0.85[d]	2+
TOX3	0.28	-0.25	-0.08	-0.08	-0.13	0.15	0.36[a]	
GATA4	0.25	-0.49[a]	-0.24	0.39[a]	-0.17	-0.12	0.02	
YAP1	-0.15	0.27	0.40[a]	0.11	0.01	0.94[d]	0.93[d]	2+
ZBTB16	0.32	0.52[b]	-0.22	-0.07	0.52[c]	0.80[d]	0.84[d]	4+
IRF1	0.52[a]	0.67[c]	-0.01	0.10	0.52[c]	0.10	0.17	2+
NEIL2	0.31	-0.11	0.28	0.39[a]	0.20	-0.22	-0.33	
RAD50	0.59[b]	0.21	0.42[a]	0.35	0.27	0.69[d]	0.51[b]	3+
KRR1	0.16	0.39	0.40[a]	0.16	0.41[a]	0.56[c]	0.56[c]	2+
RAB5B	0.29	0.20	0.36[a]	0.07	0.49[b]	0.26	0.09	1+
ARL14EP	0.18	0.53[b]	0.27	0.31	0.15	-0.06	-0.21	1+
DENND1A	0.19	-0.30	-0.12	-0.28	-0.29	-0.36[a]	-0.02	
THADA	0.27	-0.06	0.42[a]	-0.16	0.03	0.87[d]	0.86[d]	2+
MAPRE1	0.13	-0.35	0.38[a]	0.09	0.28	0.53[b]	0.59[c]	2+
AOPEP	-0.16	0.09	-0.39[a]	-0.36	-0.23	0.57[c]	0.83[d]	2+
SUOX	-0.09	-0.31	-0.32	-0.26	-0.21	-0.08	0.45[a]	
SUMO1P1	-0.08	0.10	0.17	0.59[b]	-0.20	-	-0.15	1+
FBN3	-0.34	-0.62[c]	0.03	-0.02	-0.02	0.07	-0.07	1-

Positive and negative correlations are marked in pink and blue, respectively. The colour intensity corresponds with the strength of the correlation. *P*-values: a < 0.05; b < 0.01; c < 0.001; d < 0.0001. Tissues with $P < 0.01$ were regarded as significant. [†]Number of organs with significant ($P < 0.01$) positive (+) or negative (-) correlations. '-' and '*' indicate no gene expression and correlation between same gene, respectively.

Table S28

Pearson's correlation coefficients (R) between mRNA expression levels of *AMH* and PCOS candidate genes in fetal organs and the number of organs with significant ($P < 0.01$) correlations.

Genes	Ovary (N=18)	Testis (N=27)	Heart (N=38)	Kidney (N=29)	Liver (N=37)	Cerebellum (N=33)	Brain (N=32)	Number of organs[†]
age (days)	-0.17	0.00	-0.33	-0.55[b]	-0.25	0.04	-0.34	1-
FSHB	-	-	-	-0.11	-	0.07	-	
FSHR	0.35	0.40[a]	-0.35[a]	0.05	0.03	-0.35[a]	-0.08	
LHCGR	-0.17	0.65[c]	-0.2	0.36	-0.05	-0.34	-0.02	2+
AR	-0.26	-0.34	-0.36[a]	-0.06	-0.27	-0.43[a]	0.07	
AMH	*	*	*	*	*	*	*	
INSR	0.12	0.73[d]	-0.13	-0.16	-0.03	-0.17	-0.37[a]	1+
FDFT1	-0.50[a]	0.61[c]	-0.33	0.23	-0.41[a]	0.14	0.27	1+
ERBB3	-0.22	0.59[b]	-0.34[a]	-0.55[b]	0.09	-0.38[a]	-0.22	1+, 1-
ERBB4	-0.37	-0.48[a]	-0.43[a]	-0.77[d]	0.04	-0.31	-0.14	1-
PLGRKT	0.37	0.61[c]	-0.02	-0.67[c]	-0.35[a]	0.45[b]	0.48[b]	3+, 1-
HMGA2	-0.16	-0.55[b]	-0.12	0.53[b]	-0.03	-0.33	-0.04	1+, 1-
TOX3	-0.13	-0.69[d]	-0.05	-0.60[c]	-0.19	-0.04	0.08	2-
GATA4	0.34	0.72[d]	0.09	0.18	0.11	-0.06	0.34	1+
YAP1	-0.39	-0.86[d]	-0.60[c]	-0.46[a]	-0.11	-0.40[a]	-0.10	2-
ZBTB16	-0.52[a]	-0.35	-0.42[a]	-0.12	-0.38[a]	-0.38[a]	-0.19	
IRF1	-0.25	0.09	-0.31	-0.32	-0.29	-0.34	-0.23	
NEIL2	-0.24	0.63[c]	-0.08	0.56[b]	-0.31	-0.07	-0.23	2+
RAD50	-0.67[b]	-0.56[b]	-0.64[d]	-0.33	-0.55[c]	-0.62[c]	-0.45[a]	5-
KRR1	-0.33	-0.48[a]	-0.53[b]	-0.42[a]	-0.43[b]	-0.53[b]	-0.27	3-
RAB5B	-0.76[c]	-0.34	-0.71[d]	-0.54[b]	-0.58[c]	-0.76[d]	-0.51[b]	6-
ARL14EP	-0.23	-0.38	-0.31	-0.40[a]	-0.15	-0.37[a]	-0.33	
DENND1A	0.24	0.15	-0.32	0.15	-0.22	0.02	-0.18	
THADA	-0.51[a]	-0.13	-0.59[c]	-0.46[a]	-0.27	-0.38[a]	-0.07	1-
MAPRE1	-0.55[a]	0.19	-0.31	0.05	-0.65[d]	-0.22	0.03	1-
AOPEP	0.67[b]	0.32	0.39[a]	-0.24	0.51[b]	-0.19	-0.04	2+
SUOX	0.00	0.17	0.63[d]	-0.04	0.44[b]	0.18	0.24	2+
SUMO1P1	0.32	-0.05	-0.03	-0.16	-0.27	-	-0.01	
FBN3	0.74[c]	-0.21	0.13	0.80[d]	0.07	0.09	-0.38[a]	2+

Positive and negative correlations are marked in pink and blue, respectively. The colour intensity corresponds with the strength of the correlation. *P*-values: a < 0.05; b < 0.01; c < 0.001; d < 0.0001. Tissues with $P < 0.01$ were regarded as significant. [†]Number of organs with significant ($P < 0.01$) positive (+) or negative (-) correlations. '-' and '*' indicate no gene expression and correlation between same gene, respectively.
