# Supplementary material for: Genes in loci genetically associated with polycystic ovary syndrome are dynamically expressed in human fetal gonadal, metabolic and brain tissues
Source: Front Endocrinol (Lausanne). 2023 May 8;14:1149473. doi: 10.3389/fendo.2023.1149473 (PMC10201802; doi:10.3389/fendo.2023.1149473)
Supplement: Supplementary file 3 [file Presentation_1.pdf]

Suppl Fig 1 showing expression (CPM) of RNA/DNA regulation or processing PCOS candidate genes in gonadal, metabolic and brain tissues.

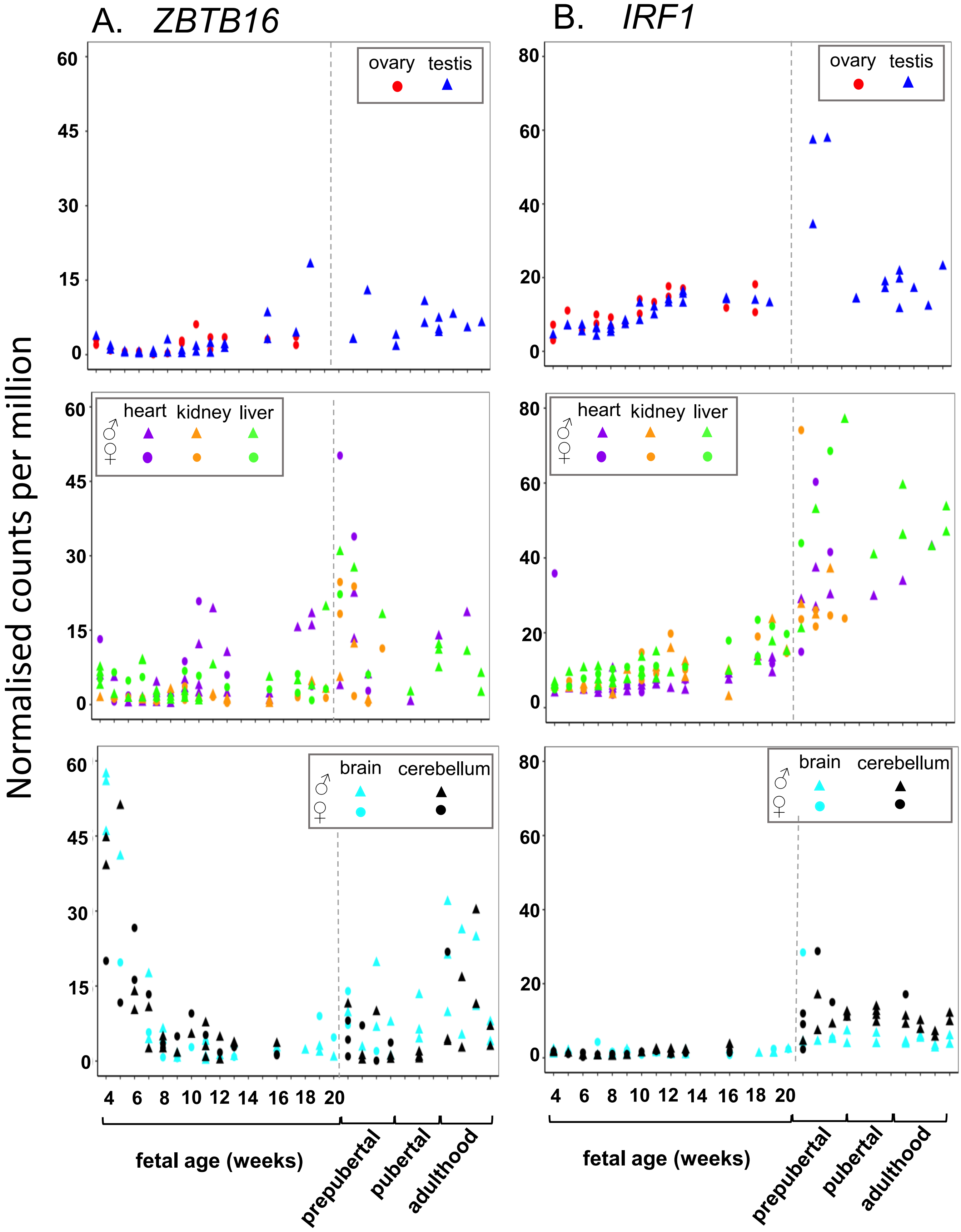

Short dashes distinguish fetal samples from the postnatal ones

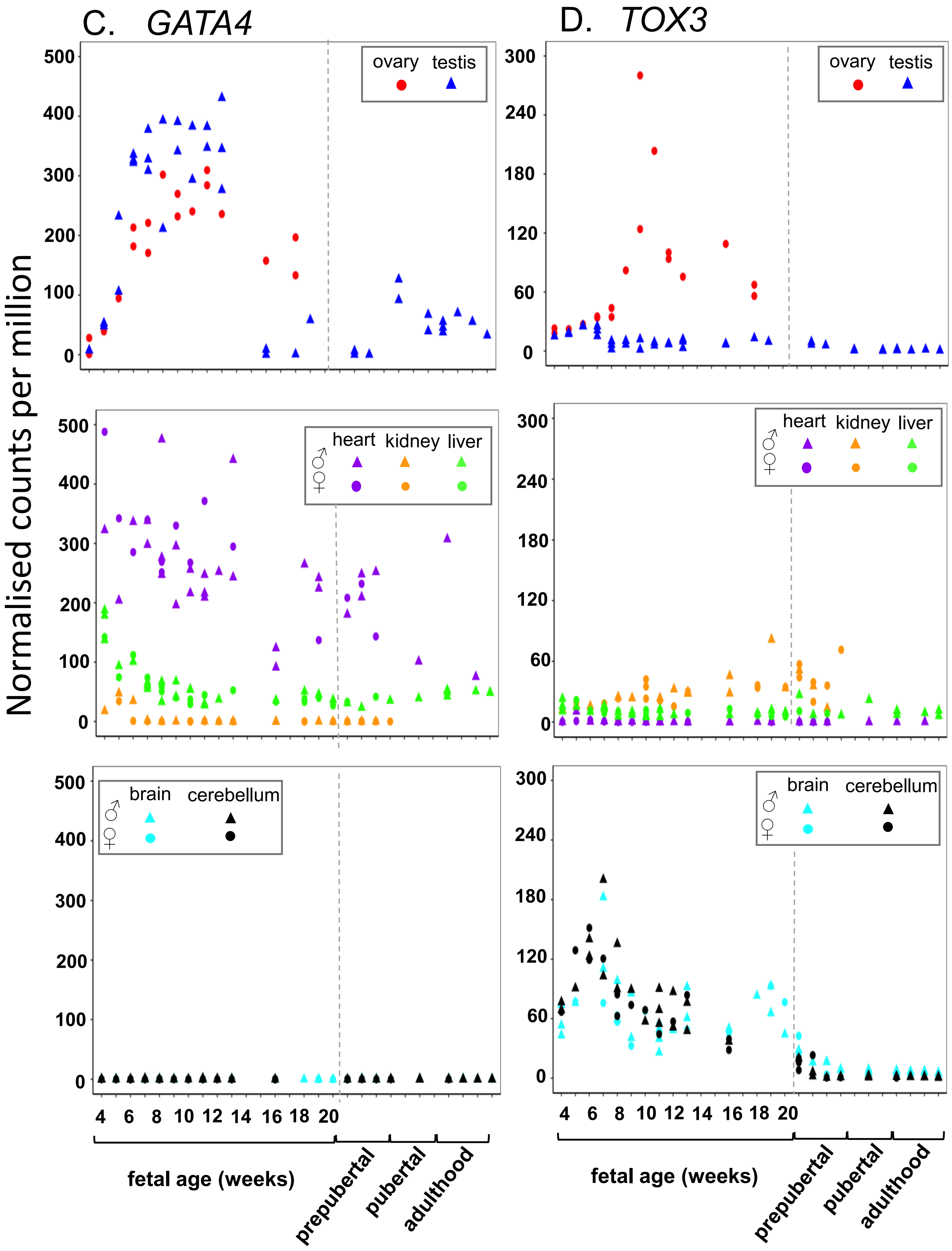

Short dashes distinguish fetal samples from the postnatal ones

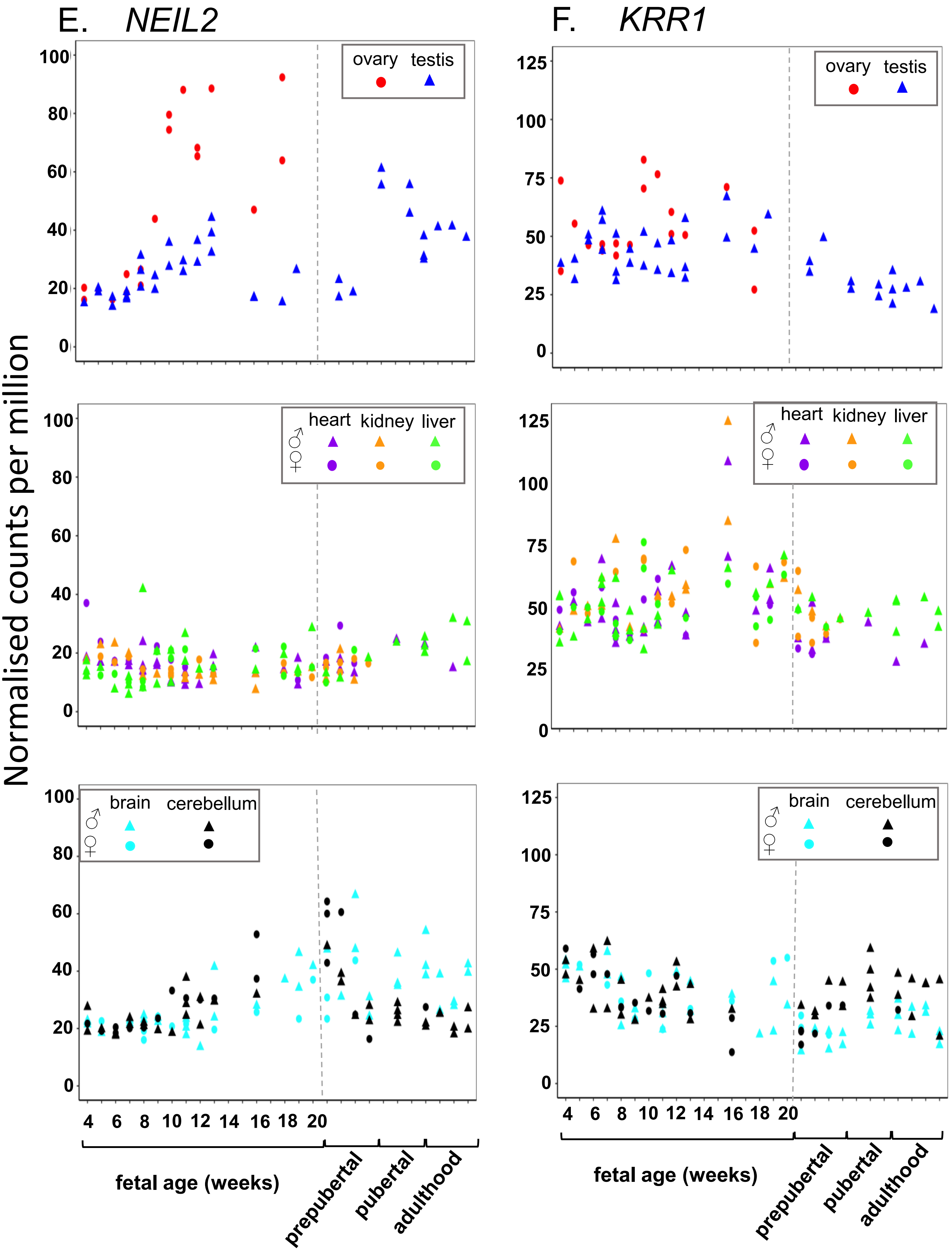

Short dashes distinguish fetal samples from the postnatal ones

Suppl Fig 2 showing expression of candidate genes involved in cell functions in gonadal, metabolic and brain tissues.

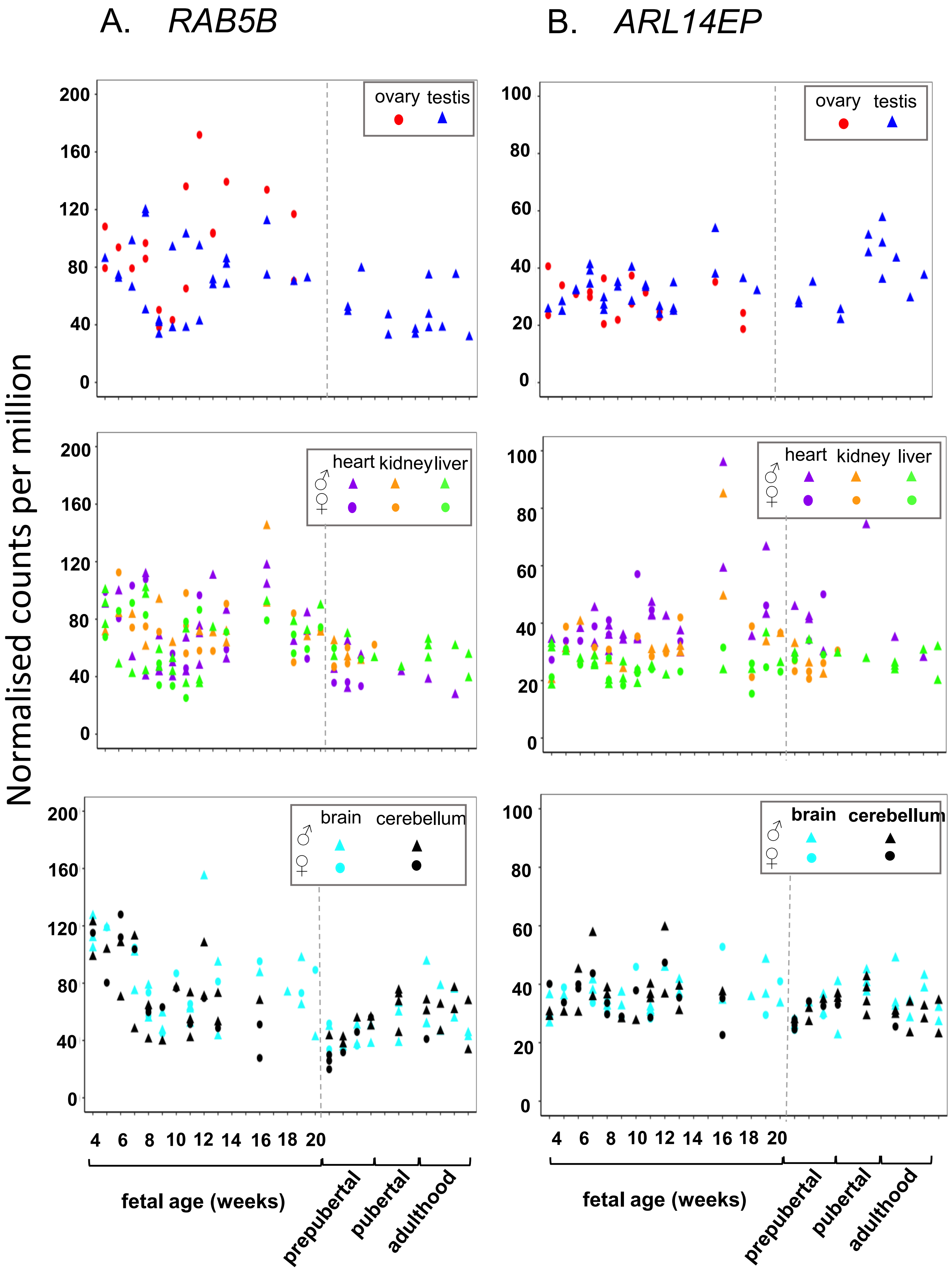

Short dashes distinguish fetal samples from the postnatal ones

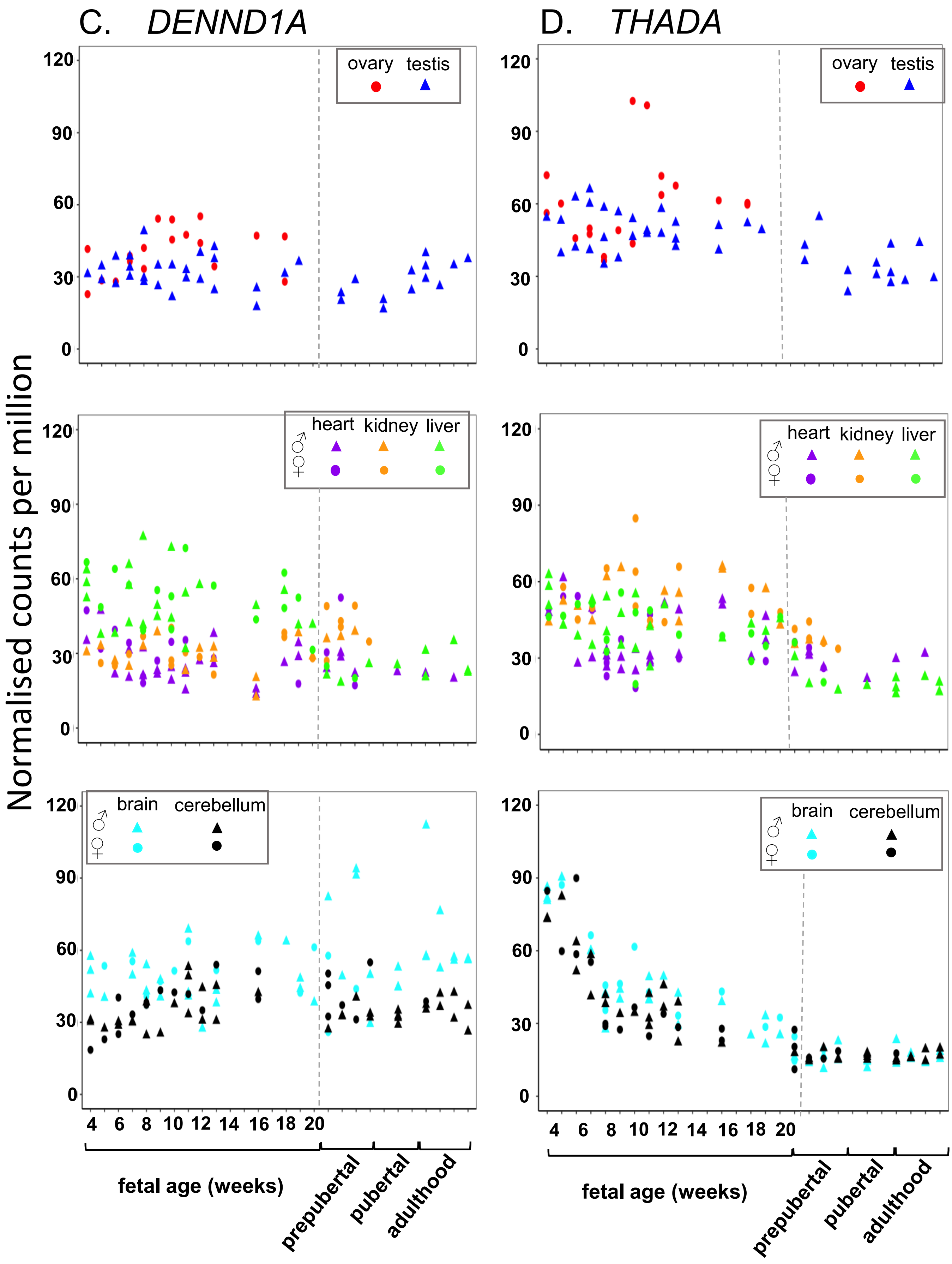

Short dashes distinguish fetal samples from the postnatal ones

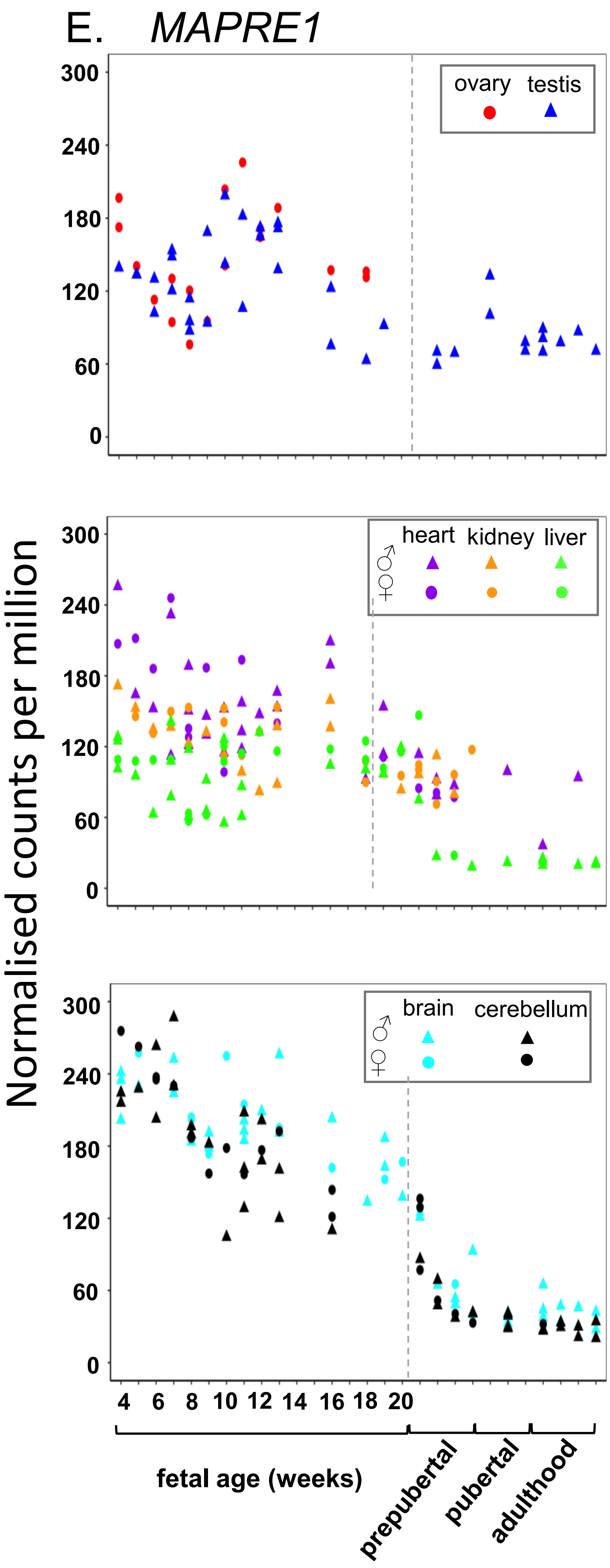

Short dashes distinguish fetal samples from the postnatal ones

Suppl Fig 3 showing expression (CPM) of enzyme-related candidate genes in gonadal, metabolic and brain tissues.

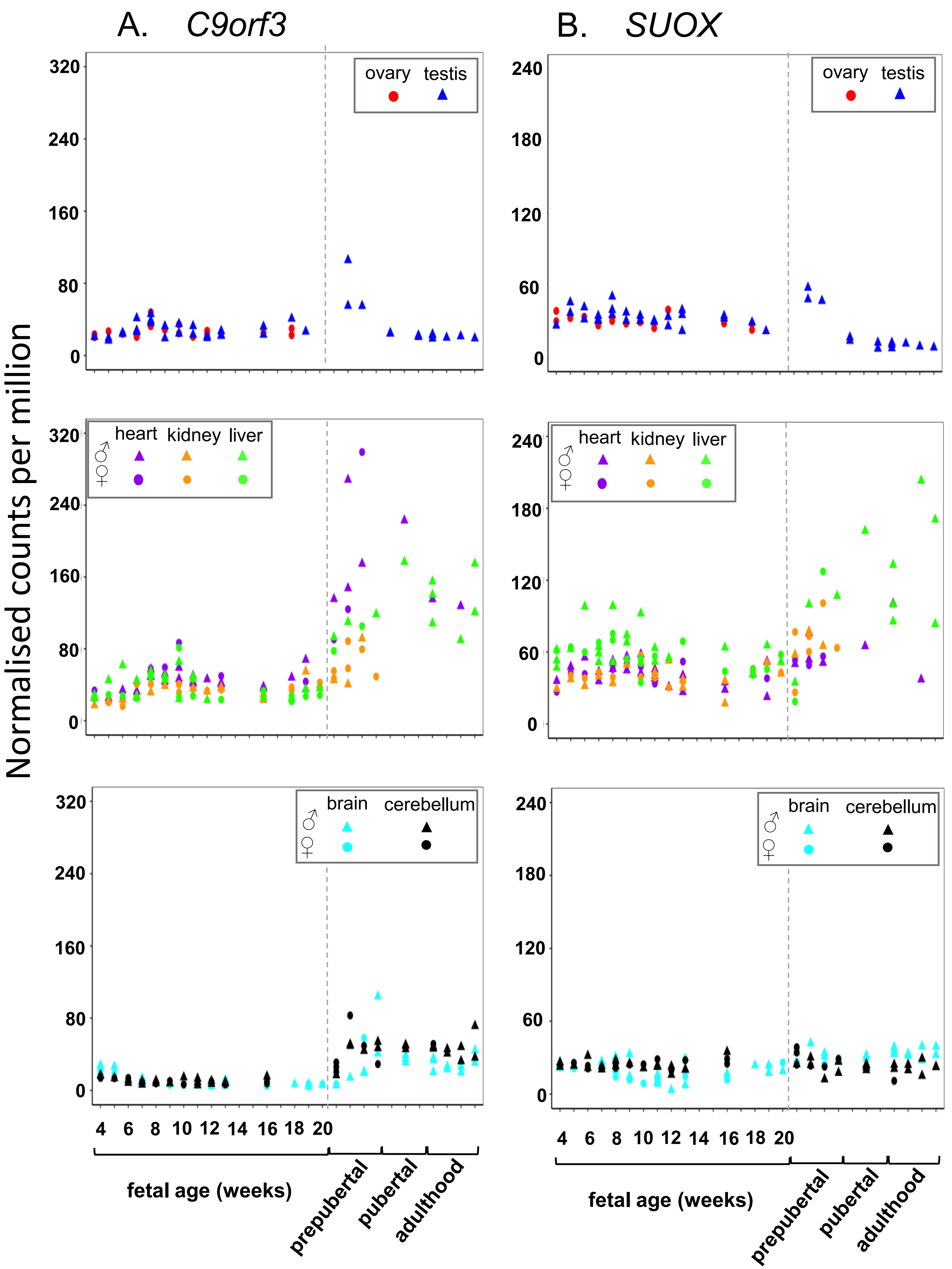

Short dashes distinguish fetal samples from the postnatal ones

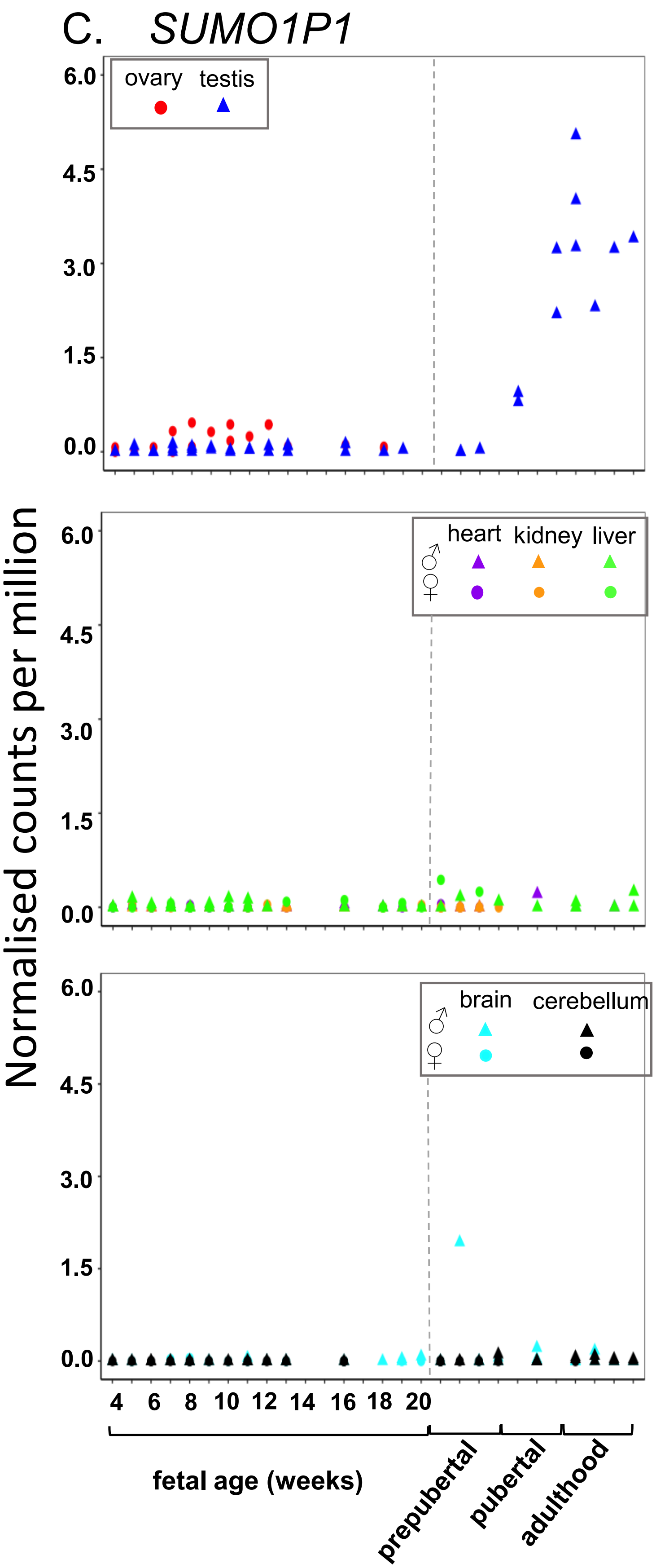

Short dashes distinguish fetal samples from the postnatal ones

Suppl Fig 4 showing expression of cell surface receptors PCOS candidate genes in gonadal, metabolic and brain tissues.

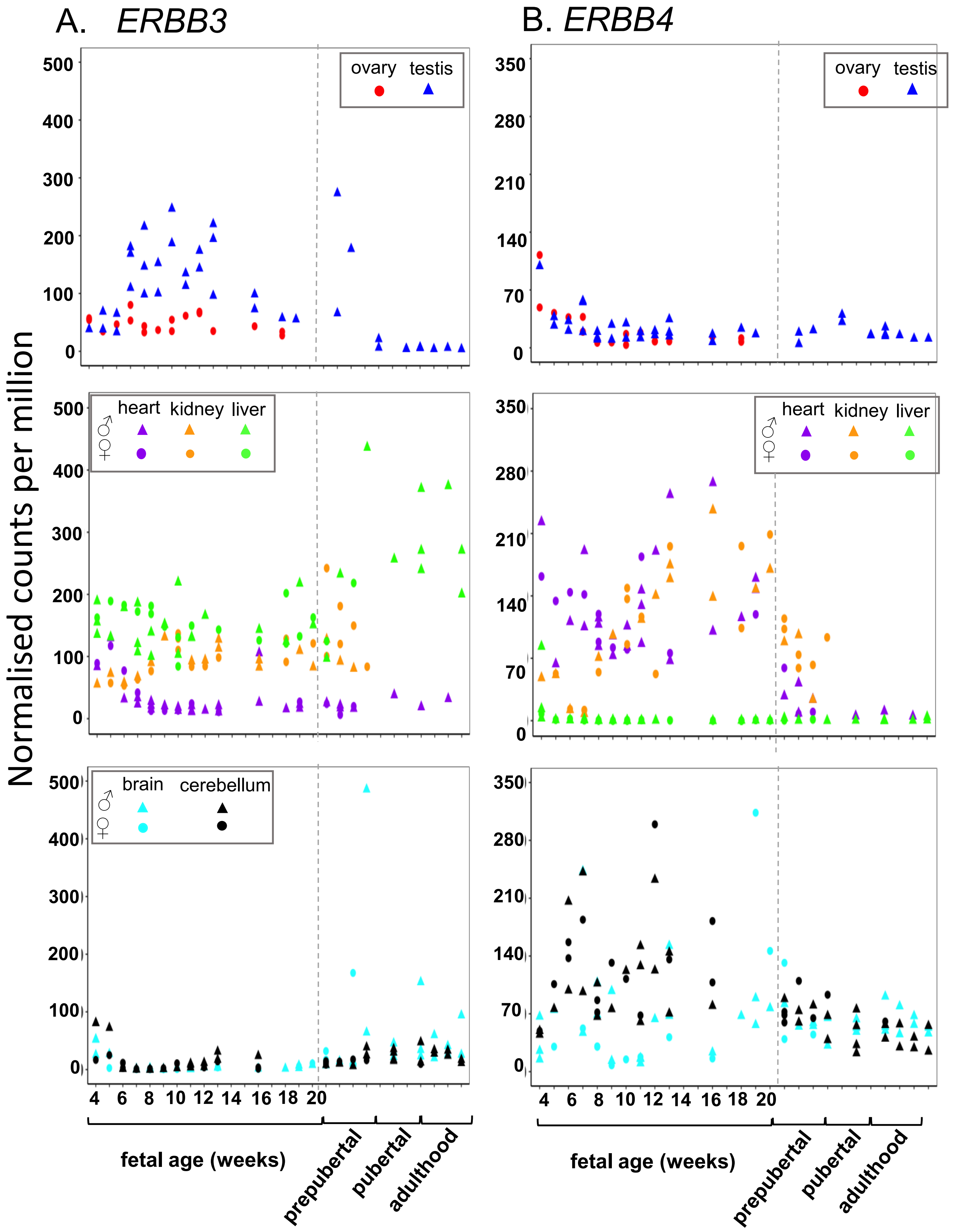

Short dashes distinguish fetal samples from the postnatal ones

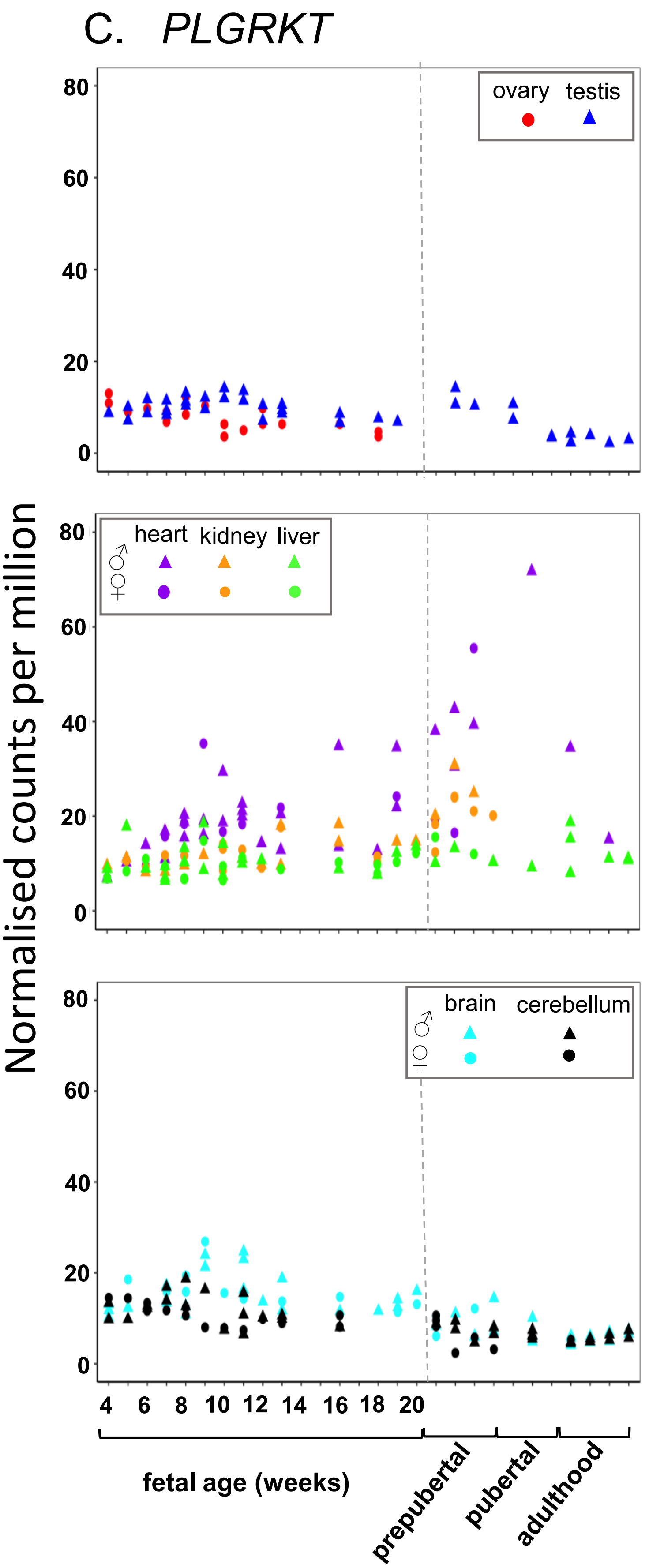

Short dashes distinguish fetal samples from the postnatal ones

Suppl Fig 5 showing expression of matrix-related PCOS candidate genes in gonadal, metabolic and brain tissues.

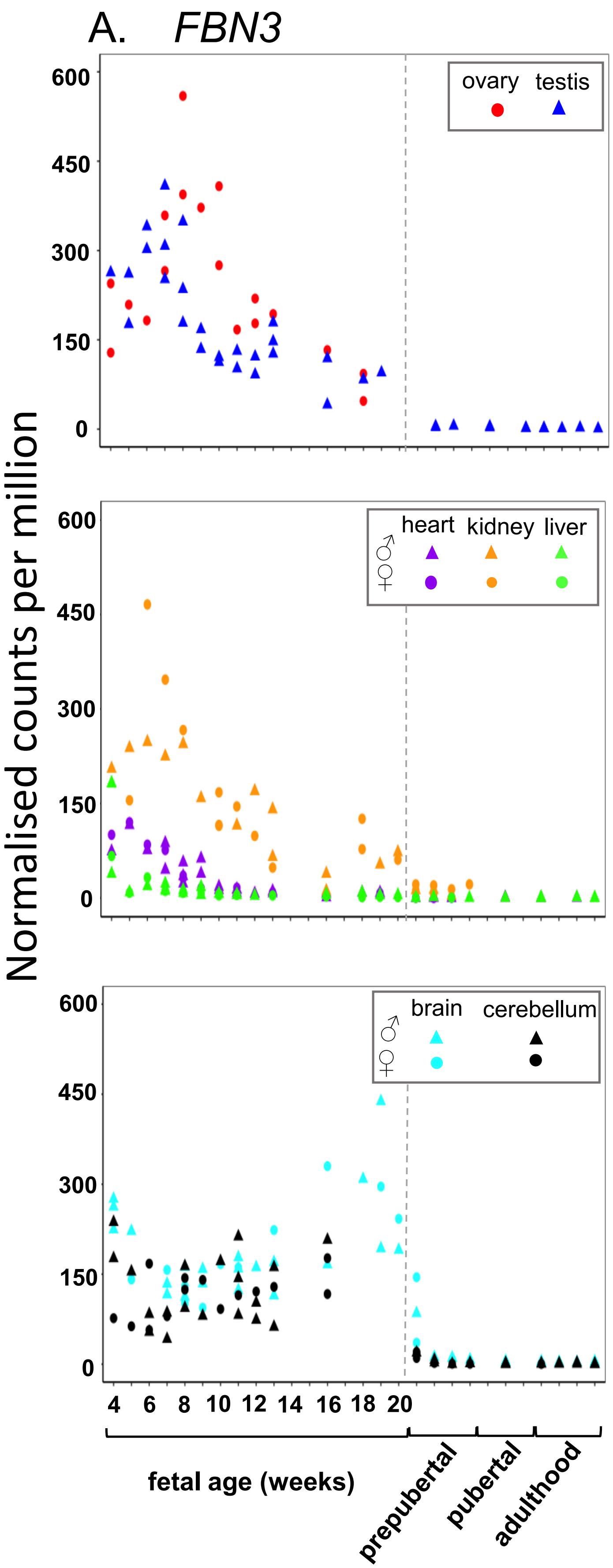

Short dashes distinguish fetal samples from the postnatal ones

Suppl Fig 6 showing expression of PCOS candidate genes involved in metabolism in gonadal, metabolic and brain tissues.

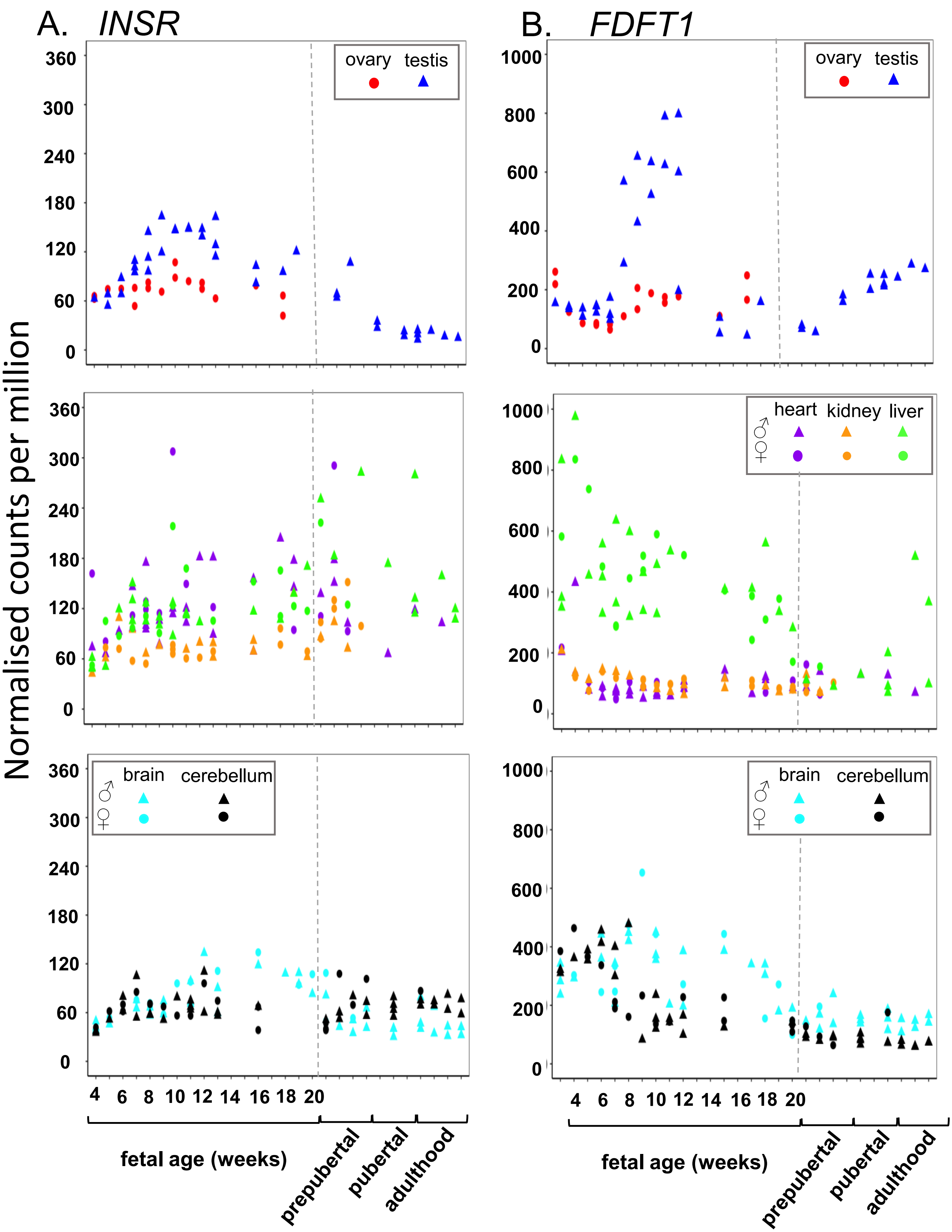

Short dashes distinguish fetal samples from the postnatal ones

Suppl Fig 7 showing expression of reproduction-related PCOS candidate genes in gonadal, metabolic and brain tissues.

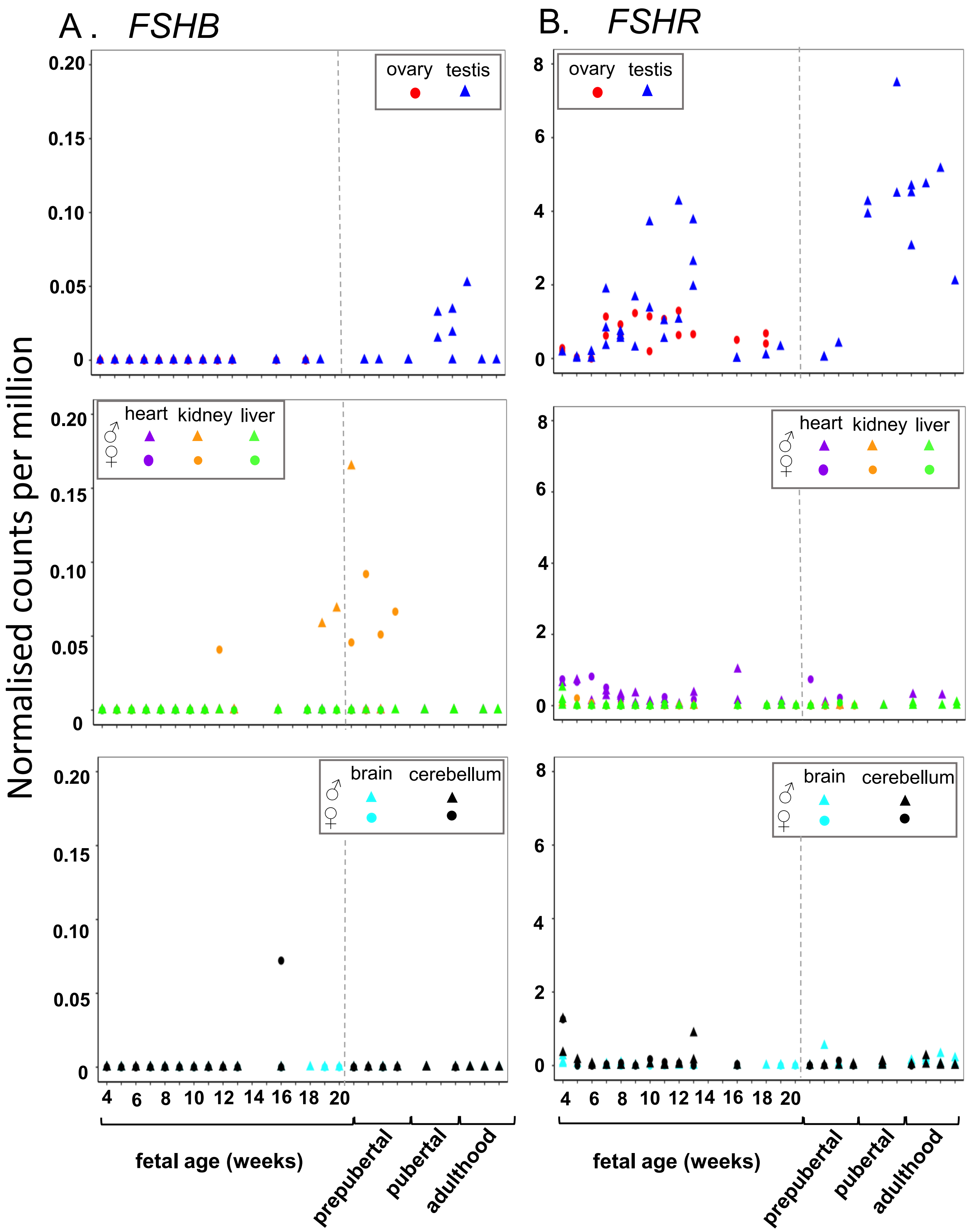

Short dashes distinguish fetal samples from the postnatal ones

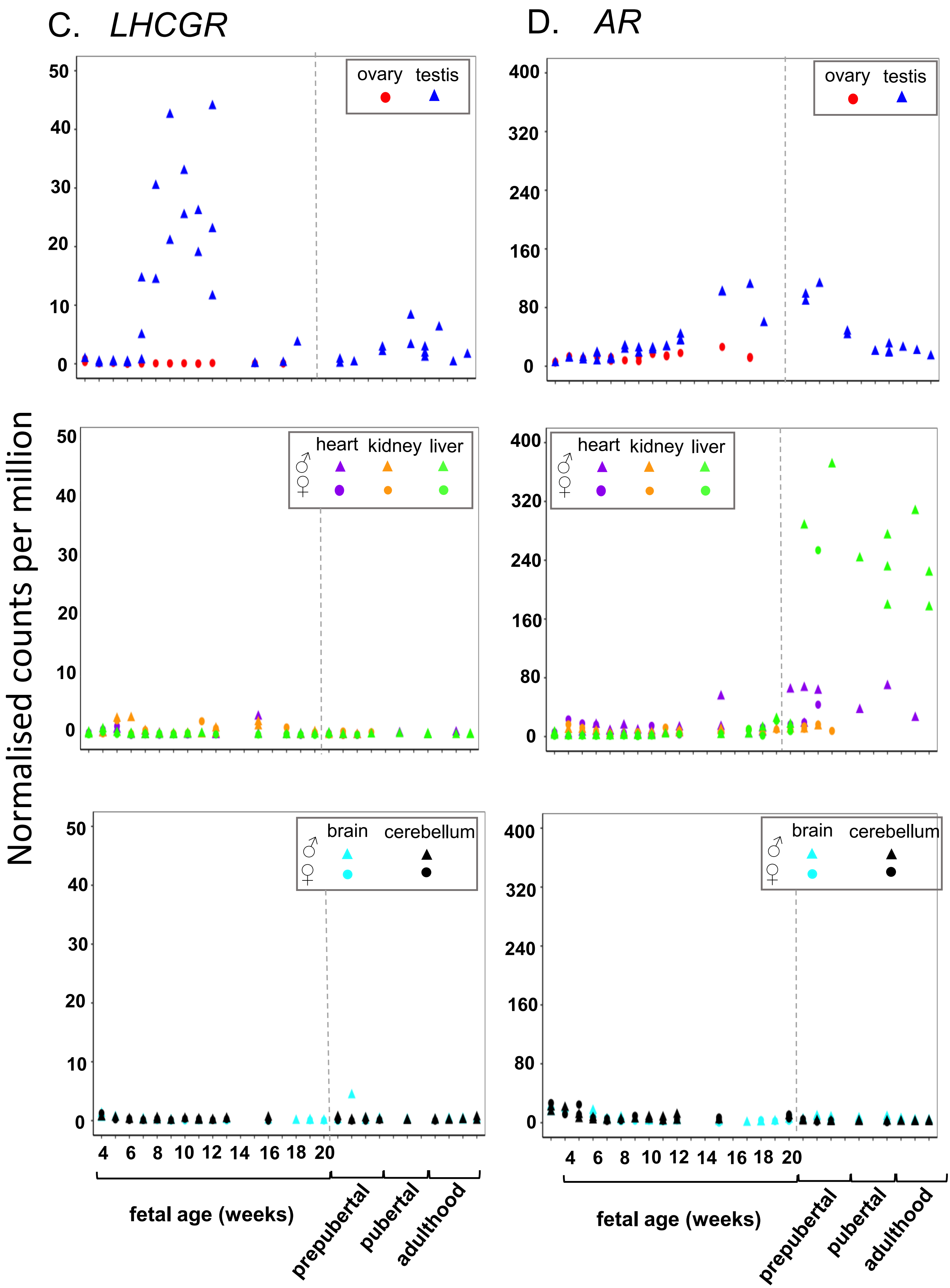

Short dashes distinguish fetal samples from the postnatal ones

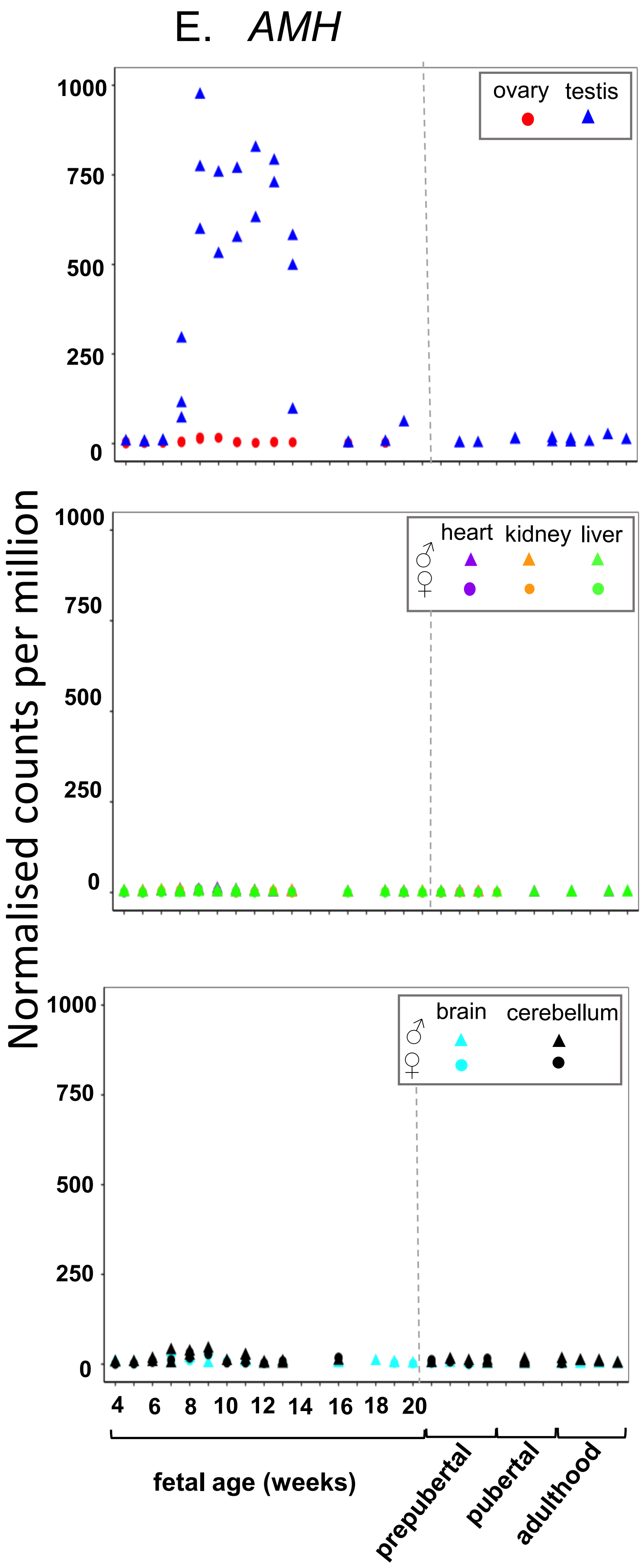

Short dashes distinguish fetal samples from the postnatal ones
